# Inhibitors of immune checkpoints—PD-1, PD-L1, CTLA-4—new opportunities for cancer patients and a new challenge for internists and general practitioners

**DOI:** 10.1007/s10555-021-09976-0

**Published:** 2021-07-08

**Authors:** Marek Z. Wojtukiewicz, Magdalena M. Rek, Kamil Karpowicz, Maria Górska, Barbara Polityńska, Anna M. Wojtukiewicz, Marcin Moniuszko, Piotr Radziwon, Stephanie C. Tucker, Kenneth V. Honn

**Affiliations:** 1 Department of Oncology, Medical University of Bialystok, Bialystok, Poland; 2 Department of Clinical Oncology, Comprehensive Cancer Center, Białystok, Poland; 3 Department of Endocrinology, Diabetology and Internal Medicine, Medical University of Bialystok, Bialystok, Poland; 4 Department of Philosophy and Human Psychology, Medical University of Białystok, Białystok, Poland; 5 Robinson College, Cambridge University, Cambridge, UK; 6 Department of Allergology and Internal Medicine, Medical University of Bialystok, Bialystok, Poland; 7 Department of Regenerative Medicine and Immune Regulation, Medical University of Bialystok, Bialystok, Poland; 8 Regional Centre for Transfusion Medicine, Bialystok, Poland; 9 Department of Hematology, Medical University of Bialystok, Bialystok, Poland; 10 Bioactive Lipids Research Program, Department of Pathology-School of Medicine, Detroit, MI USA; 11 Department of Oncology, Karmanos Cancer Institute, Detroit, MI USA; 12 Department of Chemistry, Wayne State University, Detroit, MI USA; 13 Department of Oncology, Wayne State University, Detroit, MI USA

**Keywords:** Immune checkpoint inhibitor, Immune-related adverse events, Immunotherapy, CTLA-4, PD-1, PD-L1

## Abstract

The treatment of cancer patients with immune checkpoint inhibitors (ICI) (anti-CTLA-4, anti-PD-1, anti-PD-L1, combined therapy anti-PD-1/PD-L1 with anti-CTLA-4) has without doubt been a significant breakthrough in the field of oncology in recent years and constitutes a major step forward as a novel type of immunotherapy in the treatment of cancer. ICIs have contributed to a significant improvement in the outcome of treatment and prognosis of patients with different types of malignancy. With the expansion of the use of ICIs, it is expected that caregivers will face new challenges, namely, they will have to manage the adverse side effects associated with the use of these drugs. New treatment options pose new challenges not only for oncologists but also for specialists in other clinical fields, including general practitioners (GPs). They also endorse the need for taking a holistic approach to the patient, which is a principle widely recognized in oncology and especially relevant in the case of the expanding use of ICIs, which may give rise to a wide variety of organ complications resulting from treatment. Knowledge and awareness of the spectrum of immune-related adverse events (irAEs) will allow doctors to qualify patients for treatment more appropriately, prevent complications, correctly recognize, and ultimately treat them. Additionally, patients with more non-specific symptoms would be expected, in the first instance, to consult their general practitioners, as complications may appear even after the termination of treatment and do not always proceed in line with disease progression. Dealing with any iatrogenic complications, will not only be the remit of oncologists but because of the likelihood that specific organs may be affected, is likely to extend also to specialists in various fields of internal medicine. These specialists, e.g., endocrinologists, dermatologists, pulmonologists, and gastroenterologists, are likely to receive referrals for patients suffering from specific types of adverse events or will be asked to provide care in cases requiring hospitalization of patients with complications in their field of expertise. In view of these considerations, we believe that there is an urgent need for multidisciplinary teamwork in the treatment of cancer patients undergoing immunotherapy and suffering the consequent adverse reactions to treatment.

## Introduction

Immune checkpoint inhibitors (ICI) (anti-CTLA-4, anti-PD-1, anti-PD-L1) may constitute a breakthrough in terms of a new type of immunotherapy in the treatment of cancer as they have contributed to improvement in the prognosis of patients with neoplasms, such as melanoma, non-small cell lung cancer (NSCLC), urothelial carcinoma, renal cell carcinoma, head and neck squamous cell cancer (HNSCC), or neoplasms of the lymphatic system—Hodgkin’s lymphoma. Despite their promise, however, it is only reasonable to expect that both patients and doctors will have to contend with a wide spectrum of immune-related adverse reactions associated with the treatment. Dealing with these iatrogenic complications, will not only be the remit of oncologists but because of the likelihood that specific organs will be affected, means it is likely to extend also to specialists in various fields of internal medicine. Additionally, patients with more non-specific symptoms would be expected, in the first instance, to consult their general practitioners (GPs), and thus, the effectiveness of further treatment will depend on the initial decisions taken with regard to their presentation. The most frequently reported general symptoms are fatigue and weakness, which may be a direct result of anti-PD-1/anti-CTLA-4 therapy, but may also be a symptom of endocrinopathy, e.g., hypothyroidism, or even a sign of progression of the underlying disease. Both large clinical trials and case reports serve to remind us that adverse reactions may occur at any stage of treatment, even many weeks after its completion, i.e., when the patient is no longer under close oncological supervision, but under the care of an internist or GPs [1].

The purpose of this article is an attempt to familiarize internists and GPs with the possible complications arising from the use of immune checkpoint inhibitors (PD-1/PD-L1 and CTLA-4). Antibodies targeting these pathways are designed to enhance the immune response against cancer cells. The importance of this treatment strategy is evidenced in the award of the Nobel Prize in Medicine and Physiology to J.P. Allison and T. Honjo for their contribution to the development of immunotherapy in 2018.

## Mechanisms of immune control in the process of carcinogenesis

The development of cancer is closely related to the immune system being compromised. Cancer cells can develop resistance to the mechanisms of the immune system, thus gaining the possibility of uncontrolled progression. This phenomenon can be explained in terms of cancer immunoediting theory, which contends that transformed cells may escape in the final phase of a process of control consisting of three phases: elimination, equilibrium, and escape and which constitutes a specific form of immune surveillance of cancer cells. In the first phase, suppressor mechanisms detect and eliminate developing tumors before they become clinically evident. The next step is equilibrium—a phase of tumor quiescence, in which both the tumor and immune cells are brought into a dynamic equilibrium that keeps the evolution of the cancer in check. Finally, escape represents the point of emergence of cancer cells, which either show reduced immunogenicity or trigger a large number of possible immunosuppressive mechanisms that impair the anti-tumor immune response, leading to the appearance of progressively growing tumors [2].

Immunotherapy has a well-established position in the treatment of cancer patients, especially in those with melanoma, and our state of knowledge in this area has increased significantly in recent years. While many studies have not demonstrated the expected results, current reports and experience associated with the use of interleukin-2 (IL-2) or interferon-α have shown the potential benefits that may be achieved in patients treated with therapies modulating the immune response.

In recent years, significant progress in oncology has been observed as a result of the widespread introduction of immunotherapy. Furthermore, increasing numbers of new antibodies are under clinical trials, and those already in clinical use are gaining a wider range of applications. According to data published by Jin Xin Yu et al. [3] in Nature in 2019, there is a growing interest in immuno-oncology. Over a period of 2 years (2017–2019), an increase in the number of active agents of around 91%, a 78% increase in active immuno-oncology targets, and a 60% increase in participating organizations were noted. The number of T-cell modulators used in clinical trials rose from 332 in 2017 to 620 in 2019. There can be little doubt that increasingly cancer patients worldwide will be administered immunotherapy in routine clinical settings. Table 1 presents ICIs categorized by mechanism of action that are utilized in the treatment of cancer patients.

**Table 1:** Classification of drugs according to their mechanism of action and diseases treated

CTLA-4 inhibitors	
Ipilimumab	MelanomaPediatric melanoma
Tremelimumab	Melanoma* Mesothelioma* NSCLC
PD-1 inhibitors	
Nivolumab	Melanoma NSCLC HNSCC Bladder cancer Renal cell carcinoma Hepatocellular carcinoma (HCC) Hodgkin lymphoma MSI-high, MMR-deficient metastatic colorectal cancer Cancer of the stomach, esophagus and gastro-esophageal junction*
Pembrolizumab	Melanoma NSCLC Bladder cancer HNSCC Hodgkin lymphoma Cancer of the stomach and esophagus MSI-high or MMR-deficient solid tumors of any histology Squamous cell carcinoma of the skin*
Pidilizumab	Diffuse large B-cell lymphoma (DLBCL)* Follicular lymphoma (FL)* Diffuse intrinsic pontine glioma (DIPG)* Multiple myeloma*
Cemiplimab	Squamous cell carcinoma of the skin*
PD-L1 inhibitors	
Atezolizumab	Bladder cancer NSCLC
Durvalumab	NSCLC Urothelial cancer of the bladder
Avelumab	Merkel cell carcinoma (MCC) Locally advanced/metastatic urothelial carcinoma
Combined treatment with CTLA-4 and PD-1 inhibitors	
Ipilimumab with nivolumab	Melanoma Renal cell carcinoma Cancer of the stomach, esophagus and gastro-esophageal junction*
Combined treatment with CTLA-4 and PD-L1 inhibitors	
Durvalumab with tremelimumab	Lung cancer (small cell lung cancer, NSCLC) Bladder cancer* HCC* Cancer of the head and neck area*

*Drugs undergoing clinical trials

New drugs of this kind affecting the patient’s immune system provide a challenge to doctors, not only oncologists, but also internists and GPs, who will inevitably come into contact with the adverse complications engendered by this treatment. Moreover, oncologists themselves are likely to turn to doctors of other specialties for help and support in the face of these new challenges.

## PD-1/PD-L1 axis and its role in cancer

T lymphocytes, which are responsible for inducing a specific immune response, play an important role in the immune response to an emerging antigen. Lymphocyte surface receptors are relevant ligand molecules which are stimulated when in contact with an antigen-presenting cell (APC). Cell activation requires specific recognition of the antigen presented, as well as a signal from co-stimulators that are mobilized during the formation of an immune synapse. Co-stimulators on the surface of lymphocyte cells may include the family of CD28 cell differentiation antigens [4].

Negative cell receptors are molecules that produce a signal that inhibits cell effector functions. This mechanism is designed to prevent the undesirable effects of overstimulation and ultimately cause an autoreactive response or stimulation of carcinogenesis once the defensive role of the lymphocyte antigen is terminated. This type of receptor is the PD-1 (CD279), a member of the B7 (CD28) family [5]. The transmembrane glycoprotein is expressed on activated T and B lymphocytes, natural killer (NK) cells, and monocytes. PD-1 has a cytoplasmic tail in its structure with two tyrosine kinase domains responsible for inhibitory signaling, while the expression of PD-1 during antigen stimulation is dependent on the signaling pathway of the T and B lymphocyte receptor (TCR, BCR) [6, 7].

Activation of PD-1 occurs upon binding to one of two known ligands: PD-L1 or PD-L2 [8, 9]. Each of them is expressed on the surface of APCs, including dendritic cells, but on the basis of current research, it appears that PD-L1 is mainly responsible for the suppressive effect, as it has been shown that anti-PD-1 inhibiting drugs have a greater affinity for ligands than activated T lymphocytes [10].

**Fig. 1:**
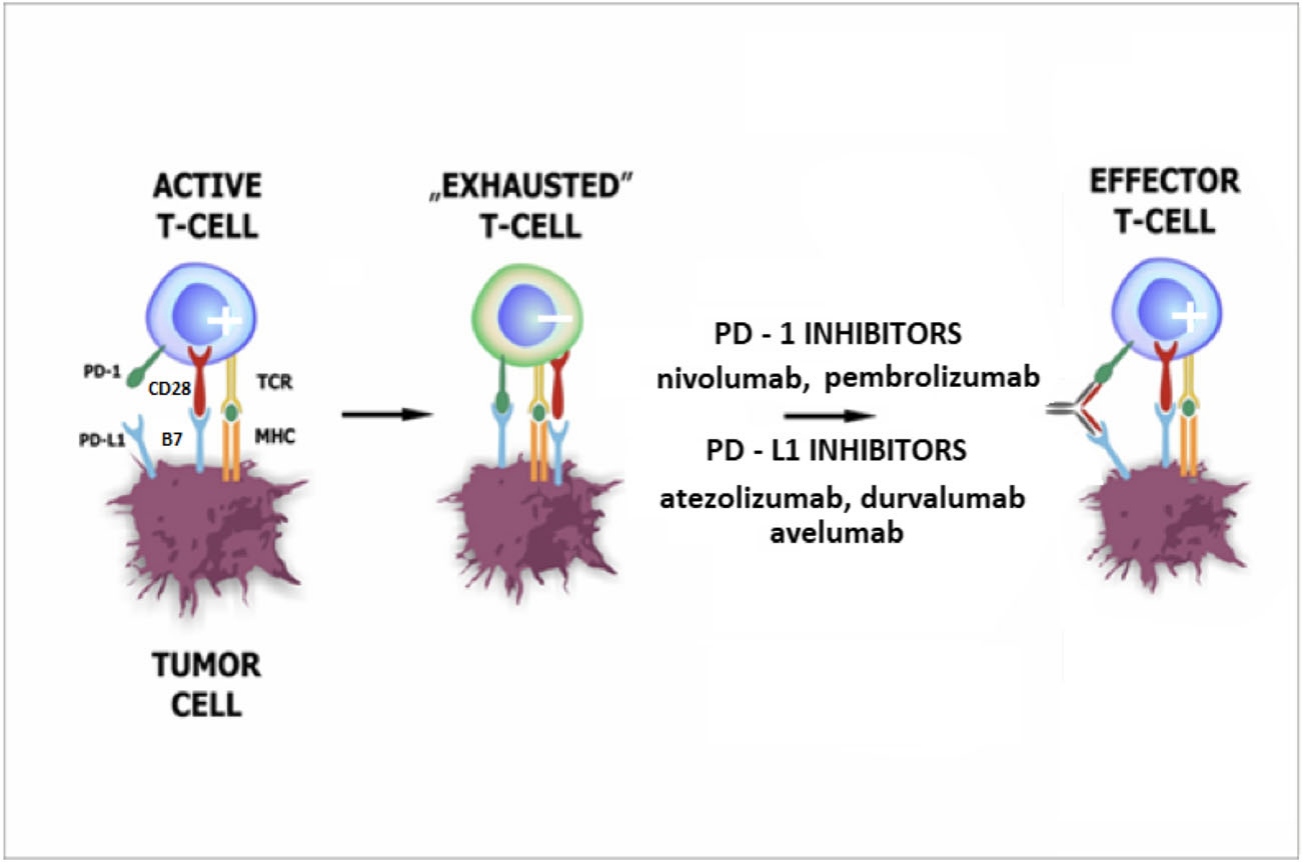
PD-1/PD-L1 axis and its inhibitors’ role in regulation of T-cell functions. During prolonged antigenic stimulation, e.g., carcinogenesis or chronic viral infections, PD-1 overexpression results in the inhibition of T-cell proliferative and cytotoxic activity. Such T-cell lymphocytes, called “exhausted” T-cells, are characterized among others by impaired ability to produce interferon γ (IFN-γ). PD-1/PD-L1 inhibitors are capable of converting “exhausted” T-cells into effector T-cells. (**+)** active T-cell, (**-)** inactive T-cell. PD-1, programmed cell death protein 1; PD-L1, programmed death-ligand 1; MHC, major histocompatibility complex

Upon activation of the PD-1 receptor by ligand signaling, the negative feedback pathway leads to TCR/BCR inhibition and a reduction in the intensity of cytokine production (in addition to inhibiting IL-10). Moreover, it impairs the production of anti-apoptotic proteins such as Bcl-2 (B-cell lymphoma) and Bcl-xL (B-cell extra-large lymphoma) [6]. The effect of PD-1 stimulation on the cell cycle has also been described. Enhancement of p15 protein expression inhibits G1 phase transition and SKP2 transcription. This gene is responsible for the coding of the protein component of ubiquitin ligase, which degrades the p27 tumor suppressor [11]. During prolonged antigenic stimulation, e.g., carcinogenesis or chronic viral infections, PD-1 overexpression leads to the T-cell phenotype described as “exhausted” whose functions are inactivated and thus reduces proliferation and the ability to produce interferon γ (IFN-γ) leading to cytotoxicity (Fig. 1). This mechanism enables the cancer cells to induce only a weak immune response, avoid elimination by the immune system, and create the conditions necessary for further development and continue the process of carcinogenesis. The intensified expression of PD-L1 on the cell surface of many types of solid tumors has been demonstrated, and numerous studies have shown that it is a negative prognostic factor in patients with melanoma, renal cell carcinoma, breast, lung, stomach, pancreas, liver, bladder, or ovarian cancer [12 - 17]. In the light of these reports, interesting observations were made in a study published in 2012, confirming that PD-L1 overexpression on the tumor surface is not always associated with a poor prognosis. Demonstrating significantly extended rates of survival in melanoma patients with confirmed exposure of PD-L1 lymphocytes makes it possible to formulate the hypothesis that their function was weakened due to the reaction with neoplastic cells by antitumor IFN-γ, which in turn led to increased PD-L1 expression. Such conclusions should be made with caution and with due attention to continuing research in this area [18].

However, the involvement of the PD-1/PD-L1 pathways as an acquired cancer adaptation mechanism is possible, and we can use this information as an indicator of resistance to the body’s defense mechanisms. The effectiveness of blocking the transduction pathway in order to achieve a better therapeutic effect in cancer patients in whom overexpression of PD-1 ligands is thought to occur by restoring the effector function of phenotypically “exhausted” T cells has been confirmed in preclinical studies. It was found that inhibition of PD-1/PD-L1 function contributes to restoration of T lymphocyte function [19] and promotes the penetration of CD8+ T cells in a mouse model of pancreatic cancer, having a synergistic effect with standard chemotherapy [20] and limiting the spread of melanoma and colorectal cancer in mice [21]. Moreover, the use of such treatment improves the effectiveness of other immunotherapy methods, including antibodies against CTLA-4 [22]. In the light of these reports, it may be concluded that antibodies blocking the PD-1/PD-L1 pathway would appear to provide appropriate indications for improving the effectiveness of immunotherapy in cancer patients.

## Anti PD-1 agents in anticancer therapy

### Nivolumab

Nivolumab is a human IgG4a antibody that was approved by the Food and Drug Administration (FDA, USA) in 2014 for monotherapy in patients with advanced or unresectable cutaneous melanoma. As the result of numerous clinical trials, the indications were extended among others to include patients with the BRAF V600 mutation who demonstrated disease progression after treatment with BRAF inhibitors [23]. In addition, combination therapy with ipilimumab was approved [24] as was the use of nivolumab in adjuvant treatment after complete surgical resection in patients with nodal involvement [25]. Cutaneous melanoma is not the only cancer in which the additional benefits of nivolumab treatment over standard chemotherapy regimens have been demonstrated. Promising treatment results have been reported in patients with progressive advanced NSCLC during or after first-line treatment with platinum-based chemotherapy. Nivolumab monotherapy was shown to be more effective than standard docetaxel chemotherapy in achieving an objective response rate (ORR) (about 20% vs 9%), extending progression free survival (PFS) and overall survival (OS) with a significantly lower rate of adverse events of G≥3 (10% vs 54%) [26]. It should be noted, however, that some studies have failed to demonstrate any extension of PFS and OS with nivolumab [27]. Nivolumab almost doubles the estimated 1-year survival in patients treated for recurrent or metastatic HNSCC after chemotherapy with platinum derivatives as compared to other drugs used in second-line treatment (docetaxel, methotrexate, cetuximab) [28]. Nivolumab monotherapy has also been shown to bring significant clinical benefit, associated with an acceptable safety profile in patients with advanced or unresectable bladder cancer who had undergone a course of chemotherapy [29].

Long-term responses to treatment and extension of overall survival were the basis for the approval of nivolumab for the treatment of patients with advanced renal cell carcinoma after prior anti-angiogenic treatment [30]. For patients with HCC, similar FDA registration decisions were driven by studies that demonstrated durable objective responses with a satisfactory safety profile [31]. In the light of recent studies, nivolumab is also an interesting therapeutic option for patients with advanced colorectal cancer with MSI or MMR deficiency [32]. This group of patients has a poor prognosis and a poor response to standard treatment, but currently available data are not yet sufficient to extend access to nivolumab for these patients, as is the case for women with advanced ovarian cancer resistant to platinum-based chemotherapy [33]. Nivolumab has been used not only in the treatment of patients with solid tumors, but also in patients with relapsed or refractory Hodgkin’s lymphoma. One study showed a high ORR of 87% and 17% of patients achieved a complete response (CR), with a slightly higher percentage of patients with complications of ≥3 degree compared to patients treated with nivolumab for solid tumors [34].

### Pembrolizumab

Pembrolizumab is a humanized antibody that, like nivolumab, belongs to the IgG4a class. Clinical trials have shown that patients diagnosed with advanced cutaneous melanoma benefit significantly from treatment with pembrolizumab in terms of response rates, extension of PFS and OS, both in treatment-naive patients [35] and in those with disease progression [36]. Pembrolizumab is also used in the first-line treatment of patients with metastatic, NSCLC, who do not have EGFR and ALK mutations and a level of neoplastic cells with PD-L1 expression in neoplastic tissue below 50% [37]. In the case of patients with a failure to respond to platinum-based chemotherapy or targeted therapy in patients with EGFR or ALK mutations, pembrolizumab can be used as the second line of treatment. Patients with NSCLC of non-squamous cell etiology may be treated with this anti-PD-1 antibody in combination with pemetrexed and platinum derivatives [38].

Pembrolizumab is also indicated in the treatment of patients with locally advanced or metastatic bladder cancer after failure of platinum-based chemotherapy or when its use is contraindicated [39, 40]. Clinical trials in patients with HNSCC have also provided promising results, though so far, the results of treatment of patients with advanced tumors have not proved sufficiently satisfactory. There is evidence that pembrolizumab monotherapy after failure of treatment with platinum-based chemotherapy regimens allows for a statistically significant extension of OS compared to standard second-line chemotherapy regimens [39]. In patients with Hodgkin’s lymphoma, pembrolizumab can be used after autologous bone marrow transplantation and brentuximab therapy or when transplantation is not possible and the patient has failed to respond to treatment with brentuximab.

### Pidilizumab

Pidilizumab is one of the first anti-PD-1 molecules to be used in cancer patients. It is a humanized, mouse IgG1 antibody that shows strong antibody-dependent cell-mediated cytotoxicity (ADCC) activity. Studies in mice have shown that T cells and NK cells are needed for the anti-tumor function to be fulfilled [41].

Phase 1 and 2 studies have been conducted to assess the efficacy of the treatment of DLBC after autologous stem cell transfer [42, 43], relapsed FL [41] and melanoma [44]. The possibility of treating patients with diffuse intrinsic pontine glioma in children [45] and with relapsed or refractory multiple myeloma (with lenalidomide) is currently being assessed.

### Cemiplimab

Cemiplimab is the first G4 antibody approved in the EU and the USA [46] for use in patients with metastatic or locally advanced cutaneous squamous cell carcinoma (CSCC). In clinical trials, half of the patients responded to treatment [46, 47]. The median value for PFS and OS [48] were not reached during the course of the research, which indicates clinically significant treatment effectiveness and durability of responses.

## Anti PD-L1 agents in anticancer therapy

### Atezolizumab

Atezolizumab is a humanized antibody indicated in monotherapy in patients with locally advanced or disseminated bladder cancer after prior platinum-based chemotherapy or with contraindications for this group of cytostatics [49]. It is also approved for the treatment of patients with locally advanced or disseminated NSCLC after prior chemotherapy or targeted treatment (depending on EGFR or ALK mutation status) [50].

### Durvalumab

Durvalumab is a human monoclonal antibody approved for the treatment of patients with locally advanced, inoperable NSCLC after radiochemotherapy. In a multicenter, randomized clinical trial, it was demonstrated that the progression-free time (17.2 vs. 5.6 months) was extended almost threefold in patients treated with durvalumab compared to placebo [51]. At the same time, in 2017, the FDA approved durvalumab by means of an accelerated procedure for the treatment of patients with locally advanced or metastatic urothelial cancer who had received no benefit from platinum-based chemotherapy [52].

### Avelumab

Avelumab is a fully human antibody that shows a double effect—it prevents the connection of PD-L1 on a tumor cell with PD-1 on T lymphocytes and has ADCC activity, which is induced by binding to receptors on the effector cells of the immune system [53, 54]. The ability of avelumab to enhance ADCC has led to a great deal of research being conducted into its mechanism of action and effectiveness in the treatment of neoplastic diseases.

Avelumab has been approved for the treatment of advanced MCC [55]. The FDA has approved avelumab as a second-line drug after or during platinum chemotherapy in locally advanced/metastatic urothelial cancer [56, 57]. After demonstrating an improvement in PFS for avelumab with axitinib as compared to sunitinib (PFS 13.8 months vs 8.4 in sunitinib) in renal cell carcinoma, it was also approved for this indication [58].

## Cytotoxic T lymphocyte-associated antigen-4 (CTLA-4) and its role in cancer

A number of studies of antibodies blocking the cytotoxic T cell antigen 4 (CTLA-4) and thus intensifying the immune response against the tumor cells have been successfully completed. Data obtained in clinical trials of ipilimumab (anti-CTLA-4) were the basis for approval of the drug in 2011 by the FDA in patients with clinically advanced melanoma.

The CTLA-4 molecule is recruited from the cytoplasm to the T cell effector membrane [59], where it forms part of the immune synapse. The activation of cytotoxic T lymphocytes takes place in two phases. The first signal is the recognition of the antigen presented by histocompatibility molecules (MHC class I or II) on the surface of APC by the TCR, which leads to an increase in the sensitivity of CD4 and CD8 receptors. The second signal necessary for synapse formation is the interaction of co-stimulating CD80/CD86 molecules (B7-1 and B7-2) on the surface of APC with CD28 on the surface of T lymphocytes, which leads to the activation and differentiation of lymphocytes. CTLA-4 competes with CD28 for binding to ligands on the APC cell, with a higher affinity for B7 family ligands, thereby displacing CD28 from association with CD80/86. The binding of CTLA-4 to ligands (CD80-B7-1, CD86-B7-2) on APC cells leads to the triggering of an inhibitory reaction-suppression of the immune response by blocking the T-lymphocyte response, reducing the proliferation of T lymphocytes, inhibiting the activity of Treg lymphocytes, and reducing cytokine secretion and consequently, to immunosuppression [59–61].

**Fig. 2:**
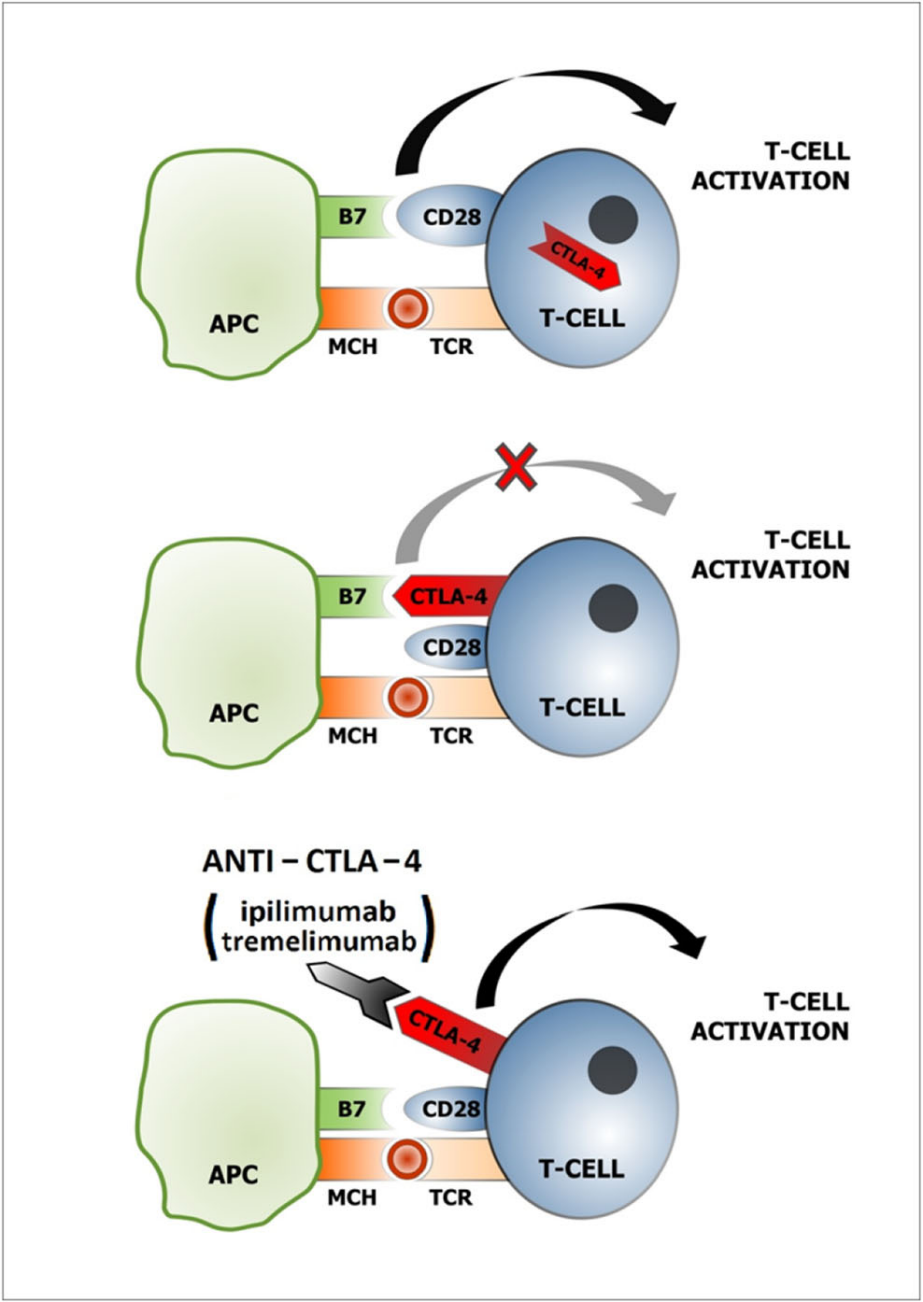
A model of T-cell activation, inhibition, and reactivation by blocking CTLA-4 with anti-CTLA-4 antibodies (ipilimumab, tremelimumab). T-cell activation requires 2 signals: the first, binding MCH with TCR; the second, interaction of CD28 on the T-cell with B7 (CD 80, CD 86) on APC. After T-cell activation, CTLA-4 is displaced to the plasma membrane and functions as a T-cell activation inhibitor. Anti-CTLA-4 antibody binds with CTLA-4 which results in T-cell reactivation. APC, antigen-presenting cell; TCR, T-cell receptor; CTLA-4, cytotoxic T lymphocyte-associated antigen 4; MHC, major histocompatibility complex

Moreover high levels of expression of CTLA-4 lead to functional reprogramming of T helper lymphocytes into regulatory T lymphocytes which exhibit strong immunosuppressive properties. All in all, CTLA-4 contributes to the immune deficiency observed in cancer patients. T-cell activation, inhibition, and reactivation by blocking CTLA-4 with anti-CTLA-4 antibodies (ipilimumab, tremelimumab) are presented in Fig. 2.

The purpose of antibody therapy is to unblock the suppressed immune response and increase the activity of T lymphocytes in the lymph nodes, which translates into an effective immune response and the destruction of neoplastic cells.

## Anti-CTLA-4 agents in anticancer therapy

### Ipilimumab

Anti-CTLA-4 was the first ICI tested with promising activity in oncological patients. In fact ipilimumab was the first antibody approved by the FDA and introduced into routine clinical practice in cancer patients. It is a fully human anti-CTLA-4 (IgG1) monoclonal antibody that has been shown to provide a long-term survival advantage in patients with advanced cutaneous melanoma [62]. Earlier, attention was also paid to the significant extension of OS, despite a relatively small percentage of objective responses to treatment (approximately 10% of patients) and the limited number of patients deriving long-term benefits from the treatment (20–25%). Attention was also drawn to the unusual profile of adverse events during the course of ipilimumab treatment—mainly skin and gastrointestinal reactions. From the very beginning, experience with the use of anti-CTLA-4 therapy led to the emphasis that patients should be under multidisciplinary medical care.

### Tremelimumab

In contrast to ipilimumab, research on tremelimumab has not yet brought promising results, which might encourage its widespread use in monotherapy [63]. In the studies published so far response rates of 15% and 30%, respectively, were reported in HCC patients treated with tremelimumab and nivolumab [64]. However, research is ongoing with regard to the combination of treatment with tremelimumab and durvalumab.

## Combined treatment with anti-PD-1/PD-L1 and anti-CTLA-4

**Fig. 3:**
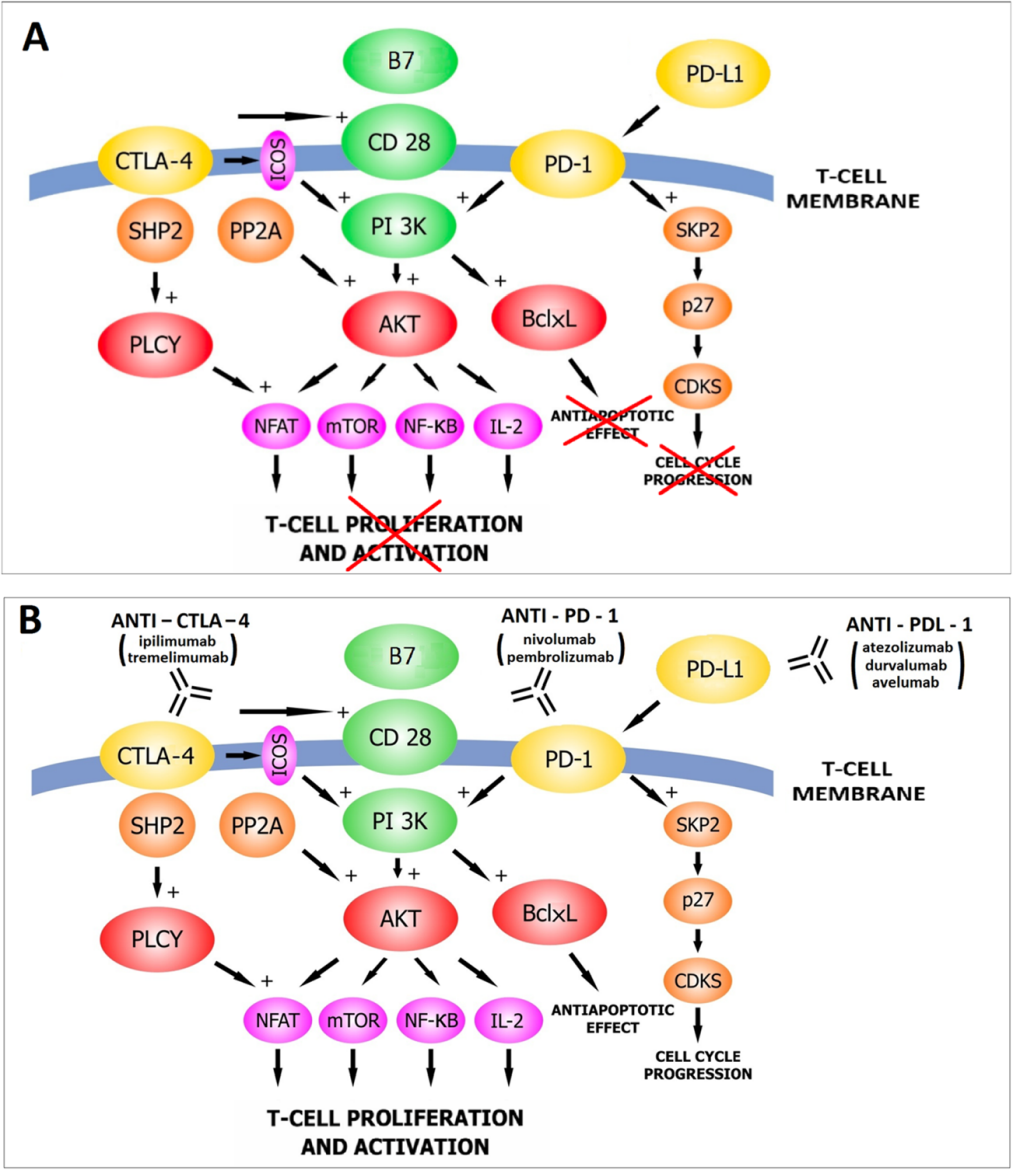
The role of immune checkpoint inhibitors (anti-PD-1, anti-PD-L1, anti-CTLA-4) in T-cell lymphocyte reactivation. Inhibited cytotoxic T lymphocyte functions in cancer patients (**A**). ICIs reactivate T-cells and thereby reinforce immunity against cancer (**B**). The use of two checkpoint inhibitors in concert (anti-PD-1/PD-L1 and anti-CTLA-4) is justified by their complementary mechanisms of action (**B**). CTLA-4, cytotoxic T-lymphocyte-associated antigen 4; Shp2, protein tyrosine phosphate 2; PLCY, phospholipase C gamma; ICOS, inducible T-cell costimulator (CD 278); PP2A, protein phosphate 2A; B7, B7-1 (CD 80), B7-2 (CD 86); PI 3K, phosphatidylinositol 3-kinase; AKT, protein kinase B; NFAT, nuclear factor of activated T-cell; mTOR, mammalian target of rapamycin; NF-KB, nuclear factor kB; IL-2, interleukin 2; BclxL, B-cell lymphoma extra-large; PD-1, programmed cell death protein 1; PD-L1, programmed death-ligand 1; SKP2, S-phase kinase-associated protein 2; p27, protein regulating cell cycle; CDKS cyclin-dependent kinases

The inhibition of two immune checkpoints has been the subject of research carried out almost in parallel with the introduction of single drugs into general use and is justified by the complementary mechanisms of action of the two (Fig. 3). While the efficacy and toxicity profile of ipilimumab treatment was known in the course of treatment of advanced cutaneous melanoma [65], the combination of anti-CTLA-4 and anti-PD-1 treatment posed new challenges in the treatment of adverse events. It was found that inhibiting two checkpoints produced better clinical outcomes than by using the drugs in monotherapy. The objective response rate for the combination of ipilimumab and nivolumab was 57.6%, while for nivolumab monotherapy it was 43.7%, compared to 19% in patients treated with ipilimumab alone for advanced skin melanoma. Moreover, the median PFS was 11.5 months, which is a significant improvement compared to ipilimumab monotherapy (2.9 months). OS was also extended with combination therapy. The 2-year OS was 64% vs 59% for nivolumab monotherapy and 45% for ipilimumab [66].

## Principles for the management of adverse events associated with the use of immunotherapy in patients with malignant tumors.

In studies published so far, particular attention has been paid to the severity, frequency, and intensity of complications arising during treatment with immune checkpoint inhibitors. In the case of monotherapy with nivolumab or ipilimumab, the incidence of adverse events was estimated to occur in around 80% of treated patients, these being mainly general symptoms of minor intensity. In the case of combination therapy, the incidence of adverse events increases to around 95% with a significant rise in the percentage of serious G3/4 adverse events (about 55%). The most common were diarrhea (44.1%), fatigue (35.1%), and itching (33.2%) [67]. In terms of gastrointestinal complications, pancreatitis and enteritis (usually diagnosed by CT) may also occur. These rare complications require termination of treatment and the introduction of immunosuppressive drugs [68].

This increased severity of adverse events is the price that patients are required to pay for improvement in the results of treatment provided by the combination of anti-CTLA-4 and anti-PD-1 in therapy. Early diagnosis and treatment of irAE lies in the hands of the health care system and medical staff, starting from the point of initial contact with GPs through to highly specialized clinics. The increase in the incidence of adverse events in G3/4 requires involvement, not only from oncologists but also specialists in other fields.

Cancer immunotherapy has now become the standard of care in many solid and hematologic malignancies. Due to their specific profile of action, their toxicity is significantly different from the adverse events of classic chemotherapy. IrAE are defined as the unique toxicity associated with the toxicity of checkpoint blockade [69]. They can be observed in about 70–90% of patients treated with ICIs [70].

With the increasing and widespread use of PD-1/PD-L1 axis inhibitors, oncologists are facing the challenge of dealing with the symptoms of overactivity of the immune system. It should be remembered that these complications often overlap with the symptoms of coexisting chronic diseases or include the occurrence of several different adverse events on the part of individual systems and organs.

The diagnosis and treatment of complications requires a special approach and specialist management. When starting the diagnosis of symptoms that have occurred as a result of immunotherapy, their etiology should always be sought and their severity determined before starting treatment. Due to the huge spectrum of adverse events, their treatment requires the cooperation of multi-specialist teams. However, patients often present in the first instance to their family doctors, who have to make the initial decisions concerning diagnosis and treatment.

Drugs used in the treatment of adverse events include glucocorticosteroids, immunomodulating drugs for which precise procedural standards have been described in the recommendations for their use and management [71]. It should also be noted that because of the effects of immunotherapy and the extended treatment periods, new complications may be expected to arise over time.

Each case of irAE should be assessed according to the National Cancer Institute’s (NCI) Common Terminology Criteria for Adverse Events (CTCAE) [72]. Adverse reactions are classified according to 5 grades, depending on the severity of symptoms: grade 1 (G1) for asymptomatic/mild, grade 2 (G2) for moderate, grade 3 (G3) for severe, grade 4 (G4) for life-threatening, and grade 5 (G5) for death. The severity of the symptoms determines any further management in terms of internal medicine and also influences the decision as to whether to continue, suspend, or discontinue further oncological treatment. The most frequent adverse events observed in cancer patients undergoing therapy with ICIs are presented in Table 2 according to their intensity as classified by NCI CTCAE. A more detailed list of AEs and their grading is available at the NCI’s website [72].

**Table 2:** National Cancer Institute’s (NCI) Common Terminology Criteria for Adverse Events v5.0 (CTCAE) [72]

Grade 1 (G1)	Grade 2 (G2)	Grade 3 (G3)	Grade 4 (G4)
Hypothyroidism			
Asymptomatic; clinical or diagnostic observations only; intervention not indicated	Symptomatic; thyroid replacement indicated; limiting instrumental ADL	Severe symptoms; limiting self-care ADL; hospitalization indicated	Life-threatening consequences; urgent intervention indicated
Hyperthyroidism			
Asymptomatic; clinical or diagnostic observations only; intervention not indicated	Symptomatic; thyroid suppression therapy indicated; limiting instrumental ADL	Severe symptoms; limiting self-care ADL; hospitalization indicated	Life-threatening consequences; urgent intervention indicated
Hypopituitarism			
Asymptomatic or mild symptoms; clinical or diagnostic observation only; intervention not indicated	Moderate; minimal, local, or noninvasive intervention indicated; limiting age-appropriate instrumental ADL	Severe or medical significant but not immediately life-threatening; hospitalization indicated; limiting self-care ADL	Life-threatening consequences; urgent intervention indicated
Adrenal insufficiency			
Asymptomatic; clinical or diagnostic observations only; intervention not indicated	Moderate symptoms; medical intervention indicated	Severe symptoms; hospitalization indicated	Life-threatening consequences; urgent intervention indicated
Colitis			
Asymptomatic; clinical or diagnostic observations only; intervention not indicated	Abdominal pain; mucus or blood in stool	Severe abdominal pain; peritoneal signs	Life-threatening consequences; urgent intervention indicated
Diarrhea			
Increase of <4 stools per day over baseline; mild increase in ostomy output compared to baseline	Increase of 4–6 stools per day over baseline; moderate increase in ostomy output compared to baseline; limiting ADL	Increase of ≥7 stools per day over baseline; hospitalization indicated; severe increase in ostomy output compared to baseline; limiting self-care ADL	Life-threatening consequences; urgent intervention indicated
Hepatic failure			
-	-	Asterixis; mild encephalopathy; drug-induced liver injury (DILI); limiting self-care ADL	Life-threatening consequences; moderate to severe encephalopathy; coma
Pneumonitis			
Asymptomatic; clinical or diagnostic observation only; intervention not indicated	Symptomatic; medical intervention indicated; limiting instrumental ADL	Severe symptoms; limiting self-care ADL; oxygen indicated	Life-threatening respiratory compromise; urgent intervention indicated (e.g., tracheotomy or intubation)
Maculopapular rash			
<10% of BSA ± symptoms (pruritus, burning, tightness)	10–30% of BSA ± symptoms; limiting instrumental ADL; >30% of BSA ± mild symptoms	>30% of BSA ±moderate/severe symptoms; limiting self-care ADL	-
Pruritus			
Mild or localized; topical intervention indicated	Widespread and intermittent; skin changes from scratching (e.g., edema, papulation, excoriations, lichenification, oozing/crusts);oral intervention indicated; limiting instrumental ADL	Widespread and constant; limiting self-care ADL or sleep; systemic corticosteroid or immunosuppressive therapy indicated	-

Certain tendencies have been observed with regard to the manifestation of individual symptoms. The first to arise are mainly skin symptoms (median 5.4 weeks from treatment initiation), followed by gastrointestinal and liver symptoms (median 7.4 weeks), and endocrine system symptoms (median 12.1 weeks). It should be noted that not all symptoms always occur nor are they of equal intensity. In so far as skin complications are concerned, they arise quite early and are frequent, so too is immunotherapy-associated pneumonia which occurs mostly at the beginning of treatment (median 3.7 weeks), but with much lower frequency and severity (a greater tendency for this complication to occur has been reported in people treated with immunotherapy due to non-squamous cell lung cancer) [73]. In the case of combined anti-PD-1 and anti-CTLA-4 therapy, symptoms of adverse events occur earlier and often with greater intensity (54% of complications in the G3/4 stage vs 16–20% for monotherapy) [69, 70]. The combination of ipilimumab and nivolumab leads to early treatment discontinuation in approximately 30% of patients. During monotherapy with anti-CTLA-4, more irAEs are observed compared to monotherapy with anti-PD-1 [69].

## Principles for the management of endocrinopathy following immunotherapy

Adverse events arising from the endocrine system are to be expected during the first 3 months of immunotherapy. The prevalence of endocrinopathy has been difficult to determine accurately due to different methods of diagnosis and monitoring used in various clinical trials. Endocrinological disorders resulting from treatment of cancer patients with ICIs are depicted in Table 3, and their frequency is presented in Table 4. Polymorphisms in the CTLA-4 gene are responsible for some autoimmune diseases, such as Hashimoto thyroiditis, type 1 diabetes, and Addison’s disease. Binding of a CTLA-4 inhibitor to specific endothelial cell surface receptors located in the endocrine glands is associated with the initiation and stimulation of an autoimmune response [82]. Clinically significant endocrinopathy occurs in less than 10% of patients treated with CTLA-4 inhibitors, but in patients treated with anti-PD -1/PD-L1, it appears to be higher. In one meta-analysis, the incidence of hypothyroidism was estimated to be 6.6% among treated patients, with a tendency for it to be higher in patients treated with anti-PD-1. Pituitary inflammation is the most common complication associated with anti-CTLA-4 treatment, while disturbances in thyroid function are observed as the most common with anti-PD-1 treatment [83, 84]. With combined treatment (anti-CTLA-4 and anti-PD-1), a higher percentage of hypothyroidism was observed compared to ipilimumab (13.2% vs 3.8%). The prevalence of hypothyroidism in all patients was independent of the type of cancer for which they were treated. Data from the meta-analysis show a significantly lower incidence of hyperthyroidism (2.5%) in all patients, with a lower incidence of ≥G3 irAE (0.1%). Hyperthyroidism was observed more frequently in patients treated with anti-PD1 than with anti-CTLA-4 or anti-PD-L1. The incidence of hyperthyroidism was significantly higher in patients treated with pembrolizumab than with nivolumab (3.8% vs 2.5%). Patients treated with combination therapy experienced this complication more frequently than with ipilimumab. The incidence of pituitary inflammation was estimated to be approximately 1.3% in all patients, but it was more frequent in patients treated for melanoma; toxicity ≥G3 was relatively rare (0.5%). Disturbances in thyroid functioning were related to the dose of ipilimumab and were more common in combined therapy with nivolumab [66].

**Table 3:** All-grade adverse events of endocrine system origin in cancer patients treated with ICI [71, 74–81]

System	Organ	Symptoms	Abnormalities in diagnostic test results	Suspected pathology
Endocrine system	Thyroid	Fatigue Weight gain Hair loss Cold intolerance Constipation Depression Bradyphrenia Weakness Decreased exercise tolerance Somnolence General slowness Feeling cold easily Cold, dry skin, Subcutaneous edema ( so called thyroid swelling) Bradycardia Hypotension Water retention Mononeuropathies Reduction in muscle strength Menstrual disorders	High TSH Low fT4 Normal/low T3 Anti-TPO (negative) Hyponatremia Hypercalcaemia USG (usually hypoechogenic)	Primary hypothyroidism
		Weight loss Increased appetite Weakness Heat intolerance Anxiety, irritability Insomnia Thyroid orbitopathy Increased sweating Palpitations Tachycardia Hypertension Diarrhea Hyperhidrosis Exophthalmos Tremors Hypermetabolic activity	Low TSH normal/high T4, T3 Thyroid-stimulating IG anti-TPO, TRAb TSI (thyroid-stimulating immunoglobulin) can be present Radioactive iodine uptake scan/technetium thyroid scan	Primary hyperthyroidism, thyrotoxicosis
	Pituitary gland	Headache Fatigue Nausea/vomiting Orthostatic hypotension Loss of libido Muscle weakness Loss of appetite Loss of weight Cold intolerance Symptoms of optic chiasm compression (visual disturbances) Anorexia	Low/normal TSH Low fT4 Hormone deficiencies (ACTH, TSH, FSH/LH) Normal/low morning cortisol Mild hyponatremia MRI (diffuse pituitary enlargement, funnel enlargement, homogeneous/heterogeneous pituitary enhancement after gadolinium administration	Hypophysitis
	Adrenal glands	Weakness Loss of appetite Muscle pain Fatigue Nausea/vomiting Weight loss Skin hyperpigmentation Abdominal pain Adrenal crisis (weakness, impaired consciousness, vomiting, diarrhea, hypotension, tachycardia, fever)	Hyponatremia Hyperkaliemia Hypoglycemia Hypercalcaemia low morning cortisol level Abnormal cortisol stimulating test Normal/high ACTH Anti-21-hydroxylase and adrenal cortex antibodies	Primary adrenal insufficiency (PAI)
	Beta cells of the pancreas	Polyuria Polydipsia Weight loss Nausea/vomiting Ketoacidosis	Glucose level Oral glucose tolerance test Lack of insulin secretion Undetectable/low C-peptide Test for antibodies (glutamic acid decarboxylase, anti-insulin, anti-islet cell A, C-peptide, zinc transporter 8)	Diabetes type 1 (insulin dependent diabetes mellitus, IDD)

**Table 4:** Incidence of all-grade endocrine adverse events in cancer patients treated with ICI [66, 71, 74–78]

Drugs/irAE	Anti-PD-1/PD-L1	Anti-CTLA-4	Combined treatment
Thyroid dysfunction	5–10% [74] 5–10% [75] 8.6–10.1% [76] 19% [77]	1–5% [74] 1–5% [75] 1.5–15.2% [76] 7% [77]	15.0% [76] 20.0% [75] 28–50% [77]
Hypothyroidism	7.0–8.3% [78] 8.6% [66]	2.8% [78] 4.2% [66]	13.2% [71] 15.0% [66] 16.3–16.4% [78]
Hyperthyroidism	3.0–3.3% [78]	0.6% [71] 0.9% [78]	8% [71] 10.2–11.1% [78]
Hypophysitis	0.4–0.7% [76] 0.5% [75] <1% [77]	1–16% [71] 2.3–6.5% [76] 2.6–4.1% [78] 3.2–17% [77] 3.9% [75]	7.7% [75] 11.7% [76]

Manifestations of endocrine inflammatory processes during immune checkpoint blockade usually involve the thyroid gland, pituitary gland, or adrenal glands [85]. Patients should be actively monitored for symptoms of endocrine disease during treatment, but there may be occasions when the patient reports symptoms to their GP. Symptoms to which doctors should be particularly alert include increased heart rate, increased sweating, extreme tiredness or weakness, muscle pain, weight gain or loss, dizziness or fainting, unusual headache, blurred vision, hunger or thirst which differs from the norm, hair loss, feeling cold, and increased frequency of urination. These non-specific symptoms are also the reason why the frequency and severity of irAE is underestimated.

Thyroid function should be monitored prior to each dose of a checkpoint inhibitor. Autoimmune thyroid disease may present as primary hypothyroidism secondary to inflammation of the thyroid gland or hyperthyroidism associated with Graves’ disease. Distinguishing primary thyroid disease from secondary hypothyroidism (usually caused by inflammation of the pituitary gland) is essential for accurate differential diagnosis. Secondary hypothyroidism usually manifests as normal or decreased TSH levels, with low FT4 levels and/or low T3 levels [76].

Acute, painless thyroiditis is the most common thyroid disorder [76]. Although less frequent, persistent primary hyperthyroidism should be treated in accordance with the usual procedures, in keeping with patients not undergoing immunotherapy. Typically, high levels of thyroid stimulating hormone (TSH) with low levels of free thyroxine (T4) indicate primary hypothyroidism, and low TSH with low free T4 indicates secondary hypothyroidism, which may be due to inflammation of the pituitary gland. Occasionally, thyroiditis with transient hyperthyroidism (low TSH and high free T4) may be followed by more longstanding hypothyroidism (high TSH and low free T4). A patient with primary hypothyroidism usually requires thyroid hormone (levothyroxine) replacement and endocrinological supervision. It is important to distinguish between hypophysitis, which is treated with steroids, from primary hypothyroidism, which is treated with hormone replacement therapy, and from sick euthyroid syndrome (normal TSH, normal free T4, and low T3-triiodothyronine), which does not require treatment. In clinical practice, a sick euthyroid syndrome (low T3) can be observed, which occurs in patients with severe, generalized debility. In the event of secondary hypothyroidism, treatment with levothyroxine should be preceded by supplementation with glucocorticoids to avoid a potential adrenal crisis.

The procedure for detecting abnormal thyroid function during routine tests depends on the type of disturbances found. In the case of an increase in TSH and normal fT4 values in asymptomatic patients, it is recommended that immunotherapy should be continued, while in the case of symptoms of hypothyroidism, it is recommended that thyroxine at TSH >10 μU/l should be introduced. In the case of an increase in TSH and low fT4 values in an asymptomatic patient, it is recommended to continue immunotherapy. If the patient reports symptoms of hypothyroidism, the recommendation is to supplement thyroxine at 0.5–1.5 μg/kg (starting with lower doses in the elderly and those with cardiac burden) and to continue cancer treatment. The detection of elevated fT4 levels in patients with normal TSH values justifies repeating tests and seeking the consultation of an endocrinologist, should abnormal results persist. Most often this is caused by taking L-thyroxine before blood sampling, so it is important that the patient does not take supplementation on the day of the examination.

On the other hand, low levels of fT4 in a patient with normal TSH values may suggest hypopituitarism and requires morning cortisol testing (9:00 am), but discontinuation of immunotherapy is not required until the diagnosis is established. If low TSH levels and low fT4 levels are found before administration of the next dose of immunotherapy, diagnosis for hypopituitarism is mandatory (MRI, morning cortisol determination). An asymptomatic patient with low TSH and elevated fT4 does not require discontinuation of immunotherapy, only the introduction of a thyreostatics and a beta-blocker in case of clinical symptoms of hyperthyroidism. Imaging diagnostics of the thyroid gland and assessment of antibodies for the TSH receptor and anti-thyroid peroxidase (anti-TPO) are also recommended. Suspending immunotherapy is indicated when the patient is unable to tolerate the symptoms of thyroid hyperactivity.

Fatigue, headache, and muscle weakness can be the clinical manifestations of hypophysitis. Less frequently reported, but also significant, are nausea, anorexia, changes in vision, and changes in mental status. These types of irAE are more common in men and elderly patients, and may occur 6 to 12 weeks after initiation of immunotherapy. The diagnosis of pituitary conditions can be all the more difficult, as a result of using steroid therapy to treat other irAEs, which may mask the symptoms of pituitary inflammation [76].

Diagnosis depends on demonstrating low levels of hormones produced by the pituitary gland. It is worth mentioning that hyponatremia may also occur, as it has been frequently reported in the case of pituitary inflammation during anti-CTLA-4 therapy [86]. Laboratory findings differentiate pituitary gland inflammation from primary adrenal insufficiency (manifested by low cortisol levels or an abnormal cortisol stimulation test and high ACTH) and primary hypothyroidism (manifested by low free T4 and high TSH). The diagnosis of pituitary gland inflammation should also be confirmed radiographically—demonstration of pituitary enlargement in MRI [87]. If pituitary inflammation is suspected, high doses of corticosteroid (1–2 mg/kg prednisone daily) administered in the acute phase may reverse the inflammatory process and prevent the need for long-term hormone therapy [76]. In most patients, however, long-term supplementation of the relevant hormones is necessary due to secondary hypothyroidism which may result in hypophysitis (treated with levothyroxine) or secondary adrenal insufficiency (treated with replacement doses of hydrocortisone—20 mg every morning and 10 mg in the evening) [88].

The most critical endocrinopathy is adrenal insufficiency with symptoms such as loss of appetite, nausea, muscle aches, abdominal pain, hypotension, dehydration, and electrolyte imbalances (hyperkalemia, hyponatremia). Progressive adrenal insufficiency leads to adrenal crisis (weakness, impaired consciousness, vomiting, diarrhea, hypotension, tachycardia, and sometimes pyrexia) and is a medical emergency. Should an adrenal crisis be suspected hospitalization is necessary and requires assessment of ACTH secretion followed by intravenous (*i.v.*) administration of corticosteroids with mineralocorticoid activity. In addition, it is essential to seek the specialist consultation of an endocrinologist, provide aggressive hydration, and ensure that evaluation for sepsis is carried out. In terms of other indications on the endocrinopathy spectrum, secondary adrenal insufficiency (low morning cortisol levels and low/normal ACTH) may also be observed.

It is worth mentioning that there have also been reports of the occurrence of fulminant type 1 diabetes mellitus (diabetes type 1, insulin dependent diabetes mellitus, IDD), which is a rare but serious and sometimes life-threatening complication. Therefore, it is important that physicians caring for patients treated with immunotherapy routinely measure blood glucose levels in their patients [89, 90].

In summary, if non-specific symptoms are encountered during immunotherapy, physicians should consider the possibility that they may signal endocrinopathies or consider specialist endocrinological consultation in order to interpret laboratory test results and guide treatment. A patient with endocrinopathy may require replacement dose steroids rather than the application of high-dose steroids. Asymptomatic endocrinopathies, such as hypothyroidism, do not require interruption or termination of immunotherapy, merely adequate supplementation and monitoring. This distinguishes these types of irAEs from others, because the endocrine organ has already sustained damaged and further immunotherapy will not result in the recurrence of clinical symptoms if the hormones concerned are supplemented. Discontinuation of treatment is only required following episodes of endocrine disruption requiring hospitalization or in the case of life-threatening conditions, e.g., adrenal insufficiency. Endocrinopathies, unlike adverse reactions in other organs/systems, may persist despite interruption or termination of immunotherapy [91]. Most often they are permanent in nature and require lifelong hormone substitution [71].

## Recommendations for the management of adverse events of gastrointestinal origin

Gastrointestinal complaints resulting from the activation of the immune system due to the use of checkpoint inhibitors are among the most common irAEs. A correlation has been observed between the occurrence of gastrointestinal irAEs in patients treated with combined anti-PD-1/CTLA-4 therapy and extended survival rates [69]. Gastrointestinal disorders resulting from treatment of cancer patients with ICIs are summarized in Table 5, and their frequency is listed in Table 6.

**Table 5:** All-grade adverse events of gastrointestinal origin in cancer patients treated with ICI [71, 74–81]

System	Organ	Symptoms	Abnormalities in diagnostic test results	Suspected pathology
Digestive system	Intestines	Diarrhea Abdominal pain Nausea Cramping Blood/ mucous in stools Changes in bowls habits Fever Abdominal distention Obstipation Constipation Dehydration Electrolyte imbalance	Blood test (anemia, elevated CRP, leukocytosis, hypoalbuminemia) Infectious workup (stool culture, Clostridium difficile, CMV serologies) Inflammatory markers (fecal leukocytes/lactoferrin/fecal calprotectin) Fecal occult blood test (FOBT) Lactoferrin—as an indicator of patients requiring urgent colonoscopy Calprotectin—shows activity of the disease Colonoscopy (normal mucosa/ mild erythema, severe inflammation with mucosal granularity, ulceration, luminal bleeding, erosions) Mucosal biopsy (lamina propria expansion, villous blunting, acute inflammation) CT imaging FDG-PET-CT	Colitis
	Liver	Yellowing of skin/whites of the eye Nausea/vomiting Pain on the right side of the abdomen Drowsiness Dark urine Bleeding or bruise more easily Feeling less hungry Fever Fatigue Malaise Hypersomnia	Elevation of serum levels of hepatic alanine/aspartate aminotransferase, GGTP, and ALP Elevated bile USG/CT/MRI Liver biopsy (portal and periportal inflammation, hepatocellular necrosis with infiltrating lymphocytes, plasma cells, and eosinophils) Coagulation disorders HIV, hepatitis A and B, blood quantiferon for tuberculosis—to prepare patients to start infliximab	Hepatitis
	Pancreas	Abdominal pain Nausea/vomiting Fever Fatigue	Increase of pancreatic enzymes (amylase, lipase) CT (swollen pancreas, reduced tissue contrast enhancement, lobulation) FDG-PET-CT (increased FDG uptake)	Pancreatitis

**Table 6:** Incidence of all-grade gastrointestinal adverse events in cancer patients treated with ICI [66, 71, 74–76, 78, 92]

Drugs/irAE	Anti-PD-1/PD-L1	Anti-CTLA-4	Combined treatment
Diarrhea	0.7–19.1% [74] 14.1–18.2% [78] 19.2% [66]	25–50% [92] 27–54% [71] 27.5–41.2% [74] 29.2% [78] 33.1% [66]	16.3–45.0% [74] 26.1–40.5% [78] 44.1% [66]
Colitis	0.3–19.1% [74] 1–5% [75] 1.3% [66] 1.8–2.1% [78] 2.2% [71]	7.6–15.5% [74] 8.0% [78] 8–22% [71] 10–25% [75] 11.6% [66]	1–13% [74] 9.2–13.4% [78] 11.8% [66] 12.8% [71] 20% [75]
Hepatitis	0.3–10.8% [74] 0.9–3.0% [78] 1–2% [71] 1.1–7.6% [76] 3.8% [75]	0.4% [78] 1.2–4.3% [76] 3–19% [71] 3.4–10.8% [74] 3.9% [75]	3.5–33% [74] 4.9–9.8% [78] 17.6% [75] 25–30% [66] 27.7% [76]

Among the gastrointestinal symptoms, the most common is immune colitis, which can manifest as diarrhea, abdominal pain, appearance of blood in feces, or perforation of the intestine. These symptoms usually manifest between the 5th and 10th week of immunotherapy (median 6–8 weeks from the start of treatment), and the symptoms usually resolve after about 4 weeks [76].

Complications in the form of diarrhea and colitis have been described in patients treated with anti-CTLA-4 antibodies. It has been estimated that diarrhea of varying degrees of severity is very common and occurs in approximately 25–50% of patients, while colitis occurs in 8–22% of those treated with ipilimumab [92]. As the incidence of these complications increases, so does their severity and gastrointestinal toxicity, rising to a level ≥ G3. These complications also constitute the most common reason for stopping treatment [93]. A relationship between the use of non-steroidal anti-inflammatory drugs (NSAIDs) and an increase in the incidence of enterocolitis during treatment with ipilimumab has also been demonstrated [94]. There have been reports of such complications even many months after the cessation of treatment [95]. Combination therapy with nivolumab/ipilimumab is reported to lead to an incidence of gastrointestinal adverse events (diarrhea, colitis) in approximately 50% of patients [66].

In all patients with symptoms of colitis (diarrhea, blood in the stools, abdominal pain), it is necessary to exclude an infectious background to such symptoms; in particular, Clostridium difficile infection should be ruled out. In such cases, symptomatic treatment should be instituted and immunotherapy continued according to clinical indications. However, painkillers should be used with caution as they may mask the symptoms of peritonitis or perforation of the intestines. During anti-CTLA-4 treatment, atypical symptoms of enterocolitis may occur, such as mouth ulcers, fistulas, abscesses, or anal fissures, as well as extra-intestinal changes (joint pain and swelling, skin lesions, hepatitis, pancreatitis). If irAEs are confirmed, the severity of symptoms should be assessed according to CTCAE. Immunotherapy can be continued with the occurrence of irAE G1 symptoms using appropriate symptomatic treatment (drugs to manage diarrhea and oral rehydration). With G2 complications, immunotherapy should be postponed, and steroid medications (e.g., prednisone 1mg/kg/day) may be started. As symptoms improve, gradual withdrawal of steroids and restitution of immunotherapy are indicated. However, if the treatment instituted does not bring clinical improvement after a minimum of 3 days, the recommended procedure for irAE G3/4 symptoms should be instigated. Endoscopic and imaging evaluations of the abdominal cavity (CT, X-ray according to indications) may be helpful in establishing the diagnosis, and it is recommended in the case of persistent G2 and G3/4 diarrhea [92].

Patients with these complications should be treated with methylprednisolone 2mg/kg (or equivalent) once or twice daily. If no improvement occurs within 3–5 days, infliximab should be administered at a dose of 5 mg/kg *i.v.* [71]; a repeat dose may be necessary after 2 weeks. Infliximab is contraindicated in the presence of intestinal perforation, sepsis, tuberculosis, or NYHA III or IV circulatory failure; therefore, tests to exclude tuberculosis, HIV, and hepatitis A and B should be performed before starting treatment. Alternative drug treatments may include mycophenolate mofetil (MMF) or tacrolimus. Long-term immunosuppression requires the inclusion of anti-infection prophylaxis in accordance with general guidelines.

New treatment strategies are also emerging. In one study, an antibody targeted against α4β7 integrin-vedolizumab was used in patients with ICI-induced colitis resistant to steroid therapy and infliximab, and remission was achieved in over 80% of patients [71].

In contrast to treatment with ipilimumab, with therapy relying on anti-PD-1/PD-L1 antibodies, the incidence and severity of gastrointestinal complications was significantly lower and usually occurred within the first 3 months of treatment [96]. A good response to treatment with corticosteroids was also seen more often. Lymphocytic enteritis is more common in patients treated with anti-PD-1, which responds well to oral budesonide treatment [97].

Hepatotoxicity associated with immunotherapy is relatively rare in patients treated with a single drug (5–10% of which only 1–2% are ≥G3). Toxicity increases significantly, however, with combination treatment of ipilimumab with nivolumab (25–30% of which about 15% are ≥G3) [66]. There was also a significant difference in the frequency of hepatotoxicity in patients treated with ipilimumab depending on the dose used (<4% for 3 mg/kg vs 15% for 10 mg/kg) [71].

In melanoma, various combinations of drugs with different mechanisms of action are commonly being tested. One such study with ipilimumab/vemurafenib (a BRAF inhibitor) was discontinued due to significant hepatotoxicity [98].

Hepatitis is usually asymptomatic and is mainly detected by laboratory tests of liver function performed before each administration of immunotherapy.

An increase in liver enzymes and/or bilirubin requires a differential diagnosis considering the potential for alcohol effects, liver metastases, viral infections, or drug interactions. In patients undergoing immunotherapy with G1 hepatic toxicity, it is possible to continue therapy while continuing to monitor liver enzymes. In the event of symptomatic G2 hepatitis, immunotherapy should be withdrawn, and the addition of steroids (prednisone 0.5–1 mg/kg) implemented if, after monitoring transaminases for 3 consecutive days, there is no spontaneous improvement. Any lack of effect following the use of steroids at this dose requires that the dose be increased to 1–2 mg/kg body weight or that it should be administered *i.v.* An increase in aminotransferases to G3/4 levels means the obligatory termination of immunotherapy and that treatment with high doses of *i.v.* steroids should be started (2 mg/kg methylprednisolone) followed by dose reduction over the next 4 weeks. If no improvement occurs or there is a recurrence of symptoms, it is possible to use an immunosuppressive drug, e.g., MMF 1g *i.v.* or 1.5g twice a day orally (*p.o*.), together with anti-infection prophylaxis. Recently, the efficacy of azathioprine (1–2 mg/kg) has also been reported in the case of failure to achieve remission with the use of glucocorticosteroids or when dose reduction is required [99].

In cases of difficult or unclear symptom etiology, liver biopsy is possible in centers specializing in hepatological diagnostics. The possibility of returning to immunotherapy after the resolution of complications in the G3 stage may be considered in terms of monotherapy, but it is not indicated for the combination of anti-CTLA-4 and anti-PD-1.

Increased levels of pancreatic enzymes, amylase and lipase, have been observed in patients receiving immune checkpoint inhibitor therapy, but symptomatic cases of pancreatitis are rare [100]. Radiographic evidence of pancreatitis with elevated enzyme levels should be considered in irAEs, and treatment with glucocorticosteroids should be initiated [76]. Disturbances in pancreatic functioning may give rise to endocrine disorders in the form of hyperglycemia or diabetes.

## Recommendations for the management of adverse events of respiratory system origin

Respiratory complications in the form of checkpoint inhibitor pneumonitis (CIP) are observed in a small percentage of patients (2–4%), although severe complications leading to respiratory failure and requiring treatment under intensive care unit (ICU) conditions are extremely rare with anti-PD-1 monotherapy. However, the frequency of such complications is almost doubled in patients treated with anti-CTLA-4 and anti-PD-1 combined therapy for melanoma [66]. Respiratory disorders resulting from treatment of cancer patients with ICIs are delineated in Table 7, and their frequency is presented in Table 8.

**Table 7:** All-grade adverse events of respiratory system origin in cancer patients treated with ICI [71, 74–77, 79–81]

System	Organ	Symptoms	Abnormalities in diagnostic test results	Suspected pathology
Respiratory system	Lungs	Flu-like symptoms New/worsening shortness of breath Dry cough Wheezing Chest pain Reduced exercise tolerance Fatigue with ADL New/increasing requirement for supplementary oxygen Dyspnea Wheezing New hypoxia Tachypnea	Blood tests (symptoms of inflammation) X-ray, HRCT/CT (progressive infiltrates and ground glass changes on lung imaging, cryptogenic organizing pneumonia, interstitial changes, pulmonary fibrosis, hypersensitivity) Decreased oxygen saturation Sputum culture Disorders in pulmonary function tests (PFTs, 6-min walk test) Bronchoscopy (inflammation) BAL (full of lymphocytes, recognition of infection) Lung biopsy (inflammatory interstitial pattern)	Checkpoint inhibitors pneumonitis (CIP)
		Dyspnea Fatigue Cough	X-ray/CT (intrathoracic lymphadenopathy, pulmonary fibrosis, nodular changes in the lungs, irregular densities) EBUS/FNA/TBBx (epithelioid non-caseating granulomas)	Sarcoidosis

**Table 8:** Incidence of all-grade respiratory adverse events in cancer patients treated with ICI [66, 74–79, 101]

Drugs/irAE	Anti-PD-1/PD-L1	Anti-CTLA-4	Combined treatment
Pneumonitis	0.4% [76] 1–5% [74] 1.4–2.0% [78] 2.7% [79] 3.8% [75]	0.4–2.2% [76] 0.7% [78] <1% [79] 7% [77]	2.1% [76] 3–7% [74] 6.5% [77] 7.5–10.5% [78] 9.6% [75] 10% [79]
Cough	4% [101]	NR	7.5% [101]
Dyspnea	3.3% [101] 4.5% [66]	4.2% [66]	9.4% [101] 10.2% [66]

*NR* not reported

It should be remembered, however, that respiratory symptoms such as coughing and shortness of breath are common, especially in people being treated for lung cancer or with metastatic lung disease. The severity of these symptoms may indicate disease progression, but it may also be a signal that further diagnostic measures are necessary to examine the possibility of complications arising from immunotherapy [77, 102].

Factors increasing the incidence of pulmonary complications in the course of immunotherapy, in addition to the presence of neoplastic changes in the lungs, include previous chest radiotherapy, COPD, advanced age [103], previous cytostatic therapy, symptomatic pneumonia, or combination therapy [77]. There is also a correlation between the incidence of pulmonary complications and the type of cancer [76]. One meta-analysis has shown a significant difference in the incidence of immunotherapy-associated CIP in patients with NSCLC compared to other cancers. CIP was observed in 3.1% of patients with NSCLC compared to 2.0% of patients with melanoma, 1.4% of patients with urothelial cancer, and 0.6% of HNSCC [104]. A better response (in terms of ORR, PFS, and OS) to anti-PD-1 immunotherapy was found in NSCLC patients with irAEs [105]. It is important to note that CIP is the leading cause of death (35–42%) among all fatalities resulting from irAEs [77].

Pneumonitis should be evaluated with imaging (preferably HRCT) and bronchoscopy with bronchoalveolar lavage (BAL), which is the preferred diagnostic option for completing sputum and blood cultures [106].

Microbiological diagnosis should be performed where immunosuppressive treatment is planned with steroids, often at high doses. Treatment of patients whose lesions have been identified only in imaging studies (as a result of limitations due to ground glass opacities, interstitial changes, hypersensitivity) is restricted in terms of postponing immunotherapy and monitoring symptoms every 2–3 days along with control investigations (such as chest X-ray, blood tests for inflammation, or sputum culture). If clinical symptoms appear, such complications are classified as G2 and antibiotic therapy should be started if an infection is suspected. Oral steroids (prednisolone 1 mg/kg/day) may be considered. If, 2 days after starting treatment, no improvement is observed clinically or in laboratory tests, the patient should be treated as having G3 side effects. In this situation, obligatory hospitalization, *i.v.* steroids (methylprednisolone 2–4 mg/kg/day), and empirical antibiotic therapy are recommended as well as HRCT and bronchoscopy with BAL. If no improvement is observed or the patient’s condition deteriorates during the next 48 h, infliximab or an MMF should be added, depending on hepatic function. At any stage of the treatment, mechanical ventilation should be considered if necessary along with admission to the ICU.

## Recommendations for the management of adverse events of musculoskeletal and rheumatological origin

Rheumatological complications are among the rarest and are observed in only 5–10% of patients [107]. However, they are more often associated with treatment using anti-PD-1 antibodies [77]. During treatment, patients may report rheumatic symptoms, which often mimic those of rheumatic diseases (including polymyalgic rheumatic diseases, rheumatoid arthritis, arthritis, myositis, vasculitis, sarcoidosis, lupus) [108]. Rheumatological and musculoskeletal disorders resulting from treatment of cancer patients with ICIs are listed in Table 9, and their frequency is listed in Table 10.

**Table 9:** All-grade adverse events of musculoskeletal and rheumatological origin in cancer patients treated with ICI [71, 74, 77–81]

System	Organ	Symptoms	Abnormalities in diagnostic test results	Suspected pathology
Musculoskeletal system	Joints	Inflammatory signs (mild)	Abnormalities in physical examination	Arthralgia/myalgia
		Swelling Pain Warmth Redness Arthralgia Stiffness after inactivity/in the morning Joint tenderness Range of motion	Physical examination (signs of inflammation) Laboratory testing (ANA, RF, anti-CCP, ESR, CRP) X-ray/USG/MRI (signs of inflammation, joint damage-erosions)	Inflammatory oligo-/polyarthritis
	Muscles	Muscle weakness Motor delay Respiratory impairment Bulbar muscle dysfunction	Normal/ elevated CK Nerve conduction study (low amplitude compound muscle action potentials/normal) EMG (irritable myopathy/normal) MRI of affected muscles Muscle biopsy	Myopathy
		Muscle inflammation Muscle weakness Muscle pain Mild myalgia Rhabdomyolysis Life treating if respiratory muscles/myocardium involved	Elevated muscles enzymes (CK) Blood testing (transaminases-AST, ALT; LDH; aldolase elevated) Inflammatory test: CRP Myositis antibody panel EMG (findings of myositis) MRI (of appropriate muscle section for biopsy) Muscle biopsy	Myositis
		Marked pain Stiffness in proximal upper and/or lower extremities No true muscle weakness Difficulty in active motion related to pain No signs of true muscle inflammation	Laboratory test (high CRP, elevated OB, anemia, thrombocytopenia, elevated liver enzymes, no CK elevation) USG/MRI (synovitis of joints and tendon sheaths)	Polymyalgia-like syndrome
		Dry eye Mouth dryness (suddenly developing, exacerbated at night) Parotitis Inflammatory myositis Inflammation of the salivary glands Thick, sticky saliva Dry throat, hoarseness Changed taste, sensitivity to spicy and sour foods	Oral mucosa changes indicating insufficient salivary gland function USG (mild changes in the major glands, including parenchymal heterogeneity with hyperechogenic bands and scattered ovoid hypoechoic lesions) Salivary gland biopsy (mild nonspecific chronic sialadenitis with acinar atrophy and fibrosis)	Sicca syndrome

**Table 10:** Incidence of all-grade musculoskeletal and rheumatological adverse events in cancer patients treated with ICI [66, 78, 80, 101]

Drugs/irAE	Anti-PD-1/PD-L1	Anti-CTLA-4	Combined treatment
Arthralgia	6.3–12.2% [78] 7.7% [66] 10% [80]	6.1% [66] 6.2–7.7% [78]	10.5% [66] 13.1–14.8% [78]
Arthritis	0.1–1.2% [78] 10% [80]	NR	0.3–0.7% [78] <1% [80]
Myalgia	3.2–5.9% [78] 3.5% [101]	3.2% [78]	5.5% [101] 6.5–11.9% [78]

*NR* not reported

The symptoms are often vague and infrequently reported as separate entities. However, in patients with a previous diagnosis of autoimmune disease, exacerbations are observed during immunotherapy [109]. NSAIDs are most commonly used in the treatment of mild to moderate rheumatic complications. In cases of limited symptom severity, intra-articular administration of steroids has been used, and in the case of greater severity, glucocorticosteroids and DMARDs have been administered.

Sicca syndrome has also been reported and observed in patients receiving checkpoint inhibitors. Symptoms develop most often within the first 3 months of treatment, often presenting suddenly with a dry mouth. Biopsy of the salivary gland shows signs of inflammation, but the picture differs from that in Sjogren’s syndrome. Glucocorticosteroids are used in the treatment, but symptoms often persist despite termination of immunotherapy [110–112].

## Recommendations for the management of adverse events of urinary system origin

Nephrotoxicity is one of the rarer complications associated with immunotherapy. Additionally, using established scales for the assessment of renal function is difficult, due to differences in the parameters assessed, e.g., between the NCI CTCAE criteria and the Kidney Disease: Improving Global Outcomes (KDIGO) acute kidney injury (AKI) classification. Hence, the decrease in eGFR is often difficult to detect [113]. Initially, renal complications were observed only in patients receiving ipilimumab (3.4%) [114]. However, these complications have also been reported in patients treated with PD-1/PD-L1 inhibitors [115, 116]. A higher incidence of renal side effects has been observed with combined anti-PD-1/PD-L1 therapy with anti-CTLA-4 at a rate of up to 5% [117, 118]. Disorders from urinary system, resulting from treatment of cancer patients with ICIs, are depicted in Table 11, and their frequency is presented in Table 12.

**Table 11:** All-grade adverse events of urinary system origin in cancer patients treated with ICI [71, 74, 76, 77, 79–81]

System	Organ	Symptoms	Abnormalities in diagnostic test results	Suspected pathology
Urinary system	Kidney	Hematuria Oliguria Hypertension Fever Eosinophilia Skin rash Weakness Loss of appetite Nausea/vomiting Oliguria	Creatine increase Eosinophilia Disorders in serum electrocytes (hyperkaliemia, mild hyponatremia) Gasometry (acidosis) Urinalysis (proteinuria, abnormal urine sediment) USG Renal biopsy (inflammatory infiltrates, involving cortex more than medulla, interstitial edema, picture generated for gel-induced interstitial nephritis, features of acute tubulointerstitial nephritis)	Nephritis, acute kidney injury (AKI)
				Acute interstitial nephritis (AIN)

**Table 12:** Incidence of all-grade urinary adverse events in cancer patients treated with ICI [74, 76, 78, 79, 81]

Drugs/irAE	Anti-PD-1/PD-L1	Anti-CTLA-4	Combined treatment
Nephritis	0.1–0.2% [78] 0.4–2% [74] 1–2% [79]	0.2% [78] 1–2% [79]	1.0–1.3% [78] 4.5% [79] 7% [74]
Renal toxicity	0.7–0.8% [78] 2% [81]	0–2.2% [76] 0.5% [78] 2% [81]	0.3–1.5% [78] 3.5% [76] 5% [81]
Acute renal failure	0.1–0.8% [78]	0.1% [78]	1.1–1.5% [81]

The most common forms of renal irAEs are acute kidney injury (AKI), which resembles drug-induced tubulointerstitial nephritis, and proteinuria [119], which can be seen from 1 to 8 months after starting treatment. Delayed reaction differentiates drug-induced toxicity, e.g., NSAIDs [113].

The diagnosis is most often made in the course of routine tests prior to the administration of subsequent doses of immunotherapy. AKI symptoms emerge much later than is usual for the drugs that normally cause kidney failure [118].

Treatment should be carried out under the watchful eye of a nephrologist, and in some cases, it would seem advisable to consider a kidney biopsy, where acute tubulointerstitial nephritis is the most common finding. In the event of G2 complications, immunotherapy should be discontinued until symptoms decrease to G1. Treatment usually involves glucocorticosteroids [76], administered orally if symptoms persist for more than a week. In G3/4, high doses of glucocorticosteroids should be used, under the supervision of a nephrologist. In the case of G3 complications, where a good treatment effect is obtained against the neoplasm, resumption of immunotherapy may be considered when reduction/resolution of toxicity is obtained.

## Recommendations for the management of adverse events of cardiovascular origin

Cardiovascular complications associated with the use of checkpoint immunotherapy are as yet largely unexplored and rare, but when they do occur, are a serious complication of treatment, often constituting a life-threatening emergency. Cardiotoxicity has been observed in the form of myocarditis and pericarditis [120], Takotsubo syndrome, arrhythmias, and vasculitis. So far, only a few such cases have been described in the literature; hence, the frequency of occurrence, predictors, and treatment are not well established. The observations to date indicate that cardiotoxicity may be one of the greatest causes of mortality among irAEs [77]. One study analyzing 88 cases found that irAEs of cardiovascular origin are characterized by elevated levels of troponins and non-specific changes in the ECG [121], which confirms the importance of performing coronary angiography during the diagnosis of cardiotoxicity associated with immunotherapy. Cardiovascular disorders resulting from treatment of cancer patients with ICIs are listed in Table 13, and their frequency is showed in Table 14.

**Table 13:** All-grade adverse events of cardiovascular origin in cancer patients treated with ICI [71,^74^, ^75^, ^77^–^80^]

System	Organ	Symptoms	Abnormalities in diagnostic test results	Suspected pathology
Cardiovascular system	Heart	Palpitations Dyspnea Chest pain Arrhythmias Pericardial/pleural effusion Acute circulatory collapse	Blood test (elevated troponin, BNP) ECG Echocardiography MRI Cardiac biopsy (features of inflammation)	Myocarditis
		Fever Chest pain on inhalation Shortness of breath Pericardial friction	Elevated biomarkers (BNP, NT-pro BNT, CK-MG, troponin) ECG (diffuse ST elevation) Echocardiography MRI	Pericarditis
		Fatigue Weakness Chest pain Palpitations Pulmonary /peripheral edema progressive/ acute dyspnea Pleural effusion Shortness of breath Irregular heartbeat Dyspnea, lack of breath	Coronarography ECG (rapid onset of heart failure, new heart block) Echocardiography CT/MRI/angiography Fasting lipid profile Elevated biomarkers (BNP, NT-pro BNT, CK-MG, troponin) Holter ECG (can show arrhythmias)	Arrhythmias Impaired ventricular function with heart failure
	Blood vessels	Symptoms of pulmonary embolism Dyspnea Pleuritic pain Cough Wheezing Hemoptysis Symptoms of deep vein thrombosis Pain Swelling Increased skin vein visibility Erythema Cyanosis accompanied by unexplained fever	Blood test (raised level of d-dimers, assessment of the coagulation system Doppler USG (positive pressure test, blood clots present in vessels) Angio-CT (visible blood clots in the lumen of the vessels)	Venous thromboembolism

**Table 14:** Incidence of all-grade cardiovascular adverse events in cancer patients treated with ICI [74, 75, 78–80]

Drugs/irAE	Anti-PD-1/PD-L1	Anti-CTLA-4	Combined treatment
Cardiotoxicity	0.06% [79] <1% [80]	NR	0.28% [79] 1.1% [78]
Myocarditis	0.1% [74] 0.5% [75]	0.1% [74]	0.3% [74] 2.4% [75]
Pericarditis	0.2–0.4% [78]	0.1% [78]	NR
Thromboembolic event	0.5–0.6% [78]	0.4% [78]	0.8–4.3% [78]

*NR* not reported

In the diagnosis of irAEs presenting in the form of cardiovascular disorders it would appear justified to use the following methods of investigation: determination of troponin levels, ECG, echocardiogram, angiography, CT, MRI, or coronary angiography. In studies on mice, it was observed that factors affecting the CTLA-4 and PD-1 axis were associated with the occurrence of autoimmune myocarditis and, consequently, the development of dilated cardiomyopathy [122, 123]. Preclinical models in patients have also indicated that maintaining proper functioning of the heart muscle is dependent on immunological checkpoints [124]. It has even been proposed that “Most cardiotoxic effects appear to be of an inflammatory nature” [125].

Myocarditis occurs in approximately 0.27–1.14% of patients. Symptoms may be typical of myocarditis, or they may resemble symptoms of acute heart failure, or arrhythmias [126], and appear within the first 30 days (median value) of therapy, often after just the first dose [127]. The risk is greater with a combined anti-PD-1/anti-CTLA-4 (at 2.4%) [128].

The irAE spectrum also includes pericarditis or temporal arteritis accompanied by the risk of blindness [127].

It is advisable that patients should be examined in cardio-oncology centers [129], because early diagnosis and the use of appropriate treatment methods can help to reduce mortality from these adverse side effects, estimated at approximately 50% [121]. This is extremely important because myocarditis appears early following the initiation of immunotherapy and is characterized by a malignant course. Improvement in the condition of patients has been observed after the use of high doses of steroids. Therefore, caring for such patients requires experienced medical personnel [120].

## Recommendations for the management of adverse events of hematological origin

Autoimmune hemolytic anemia has been reported in a patient treated with nivolumab. Other complications include red blood cell aplasia, neutropenia, thrombocytopenia, hemophilia A, multidysplastic syndrome, fatal anaplastic anemia, and immune thrombocytopenic purpura [77]. Significant improvement was observed after discontinuation of immunotherapy and introduction of glucocorticosteroids. The most common hematological adverse events are described in Table 15, and their frequency is presented in Table 16.

**Table 15:** All-grade adverse events from hematological origin in cancer patients treated with ICI [71, 74–80]

System	Symptoms	Abnormalities in diagnostic test results	Suspected pathology
Hematological	Weakness Paleness Jaundice Dark-colored urine Fever Inability to do physical activities Heart murmur	Blood test (macrocytosis, elevated unconjugated bilirubin, LDH,reticulocyte count, reduced haptoglobin in serum) Peripheral blood smear (spherocytosis) Direct Coombs test positive for IgG/C3 Bone marrow biopsy	Autoimmune hemolytic anemia
	Macroangiopathic hemolytic anemia Thrombocytopenic purpura Fever Renal function abnormalities Neurologic abnormalities (seizures, hemiplegia,visual disturbances)	Blood test (low hemoglobin, low platelets) Renal function abnormalities	Acquired thrombotic thrombocytopenicpurpura (acquired TTP)
	Thrombotic microangiopathy Renal failure Hemolytic anemia Bloody diarrhea Decreased urination Blood in urine Pallor Small, unexplained bruises Bleeding from nose/mouth Fatigue, irritability Confusion/seizures High blood pressure Swelling of the face, hands, feet or entire body Abdominal pain Vomiting	Blood test (low level of RBC/hemoglobin, erythroblast/schistocytespresent, increased level of reticulocytes, elevated free bilirubin,increased level of LDH, severe thrombocytopenia) Urinalysis (proteinuria, hematuria)	Hemolytic uremic syndrome (HUS)
	Symptoms of anemia	Peripheral blood smear (pancytopenia with scattered lymphocytes) Coombs test Reticulocyte count Hemolysis assays (LDH, haptoglobin, bilirubin) Bone marrow aspiration/biopsy (hypocellularity with stroll edema,no signs of fibrosis, virtual absence of hematopoietic elements,hypo-/aplasia) Flow cytometry (lymphocytes usually represent 50% of the sample,mostly CD-positive T cells)	Aplastic anemia
	Bruising easily Pinpoint-sized petechiae, often on the lower legs Spontaneous nosebleeds Bleeding from the gums (e.g., during dental work) Blood in the urine Blood in the stools Abnormally heavy menstruation Prolonged bleeding from cuts	Decreased platelet count Increased levels of platelet-associated IgG Normal white blood cell count and hemoglobin level Bone marrow biopsy (increased number of megakaryocytes with ahigh percentage of immature platelets and with abnormal cells) Antiplatelet antibodies	Immune thrombocytes purpura (ITP)
	Subcutaneous hemorrhages Mucosal bleeding (into gastrointestinal,urinary and genital tract) Bleeding into the muscles Intracranial bleeding	Elongation of APTT with normal PT, TT, platelet count, and fibrinogen Lowered factor VIII activity	Acquired hemophilia

**Table 16:** Incidence of all-grade hematological adverse events in cancer patients treated with ICI [71, 101]

Drugs/irAE	Anti-PD-1/PD-L1	Anti-CTLA-4	Combined treatment
Hematological toxicity	<1% [71]	NR	NR
Anemia	3.2% [101]	NR	3.9% [101]

*NR* not reported

## Recommendations for the management of adverse events affecting the skin

Skin complications are earliest to appear and the most common adverse reactions in connection with immunotherapy in patients treated with anti-CTLA-4 (reported in approximately 45–65% of patients treated with ipilimumab) and anti-PD1 (approximately 30–40% treated with anti-PD-1/PD-L1: nivolumab/pembrolizumab) [130, 131]. IrAEs that arose from ipilimumab appeared within 12 weeks of starting treatment [91]. Combined therapy with ipilimumab/nivolumab resulted in the occurrence of adverse events affecting the skin in over 70% of treated patients, G3/4 occurring in approximately 20% of patients [132]. Dermal toxicity in the case of anti-CTLA-4 and anti-PD-1 antibodies was manifested earlier and took a longer and more severe course [133]. Itching was the most frequently reported symptom during treatment, which was associated with maculopapular rash or with normal-looking skin [77]. Dermatological disorders resulting from treatment of cancer patients with ICIs are delineated in Table 17, and their frequency is listed in Table 18.

**Table 17:** All-grade adverse events of dermatological origin in cancer patients treated with ICI [71, 74–80]

System	Symptoms	Abnormalities in diagnostic test results	Suspected pathology
Skin	Maculopapular rash Erythema multiforme Eczematous Psoriasiform Skin rash (maculopapular lesions) Dry skin	Full skin and mucosal examination with attentionto lesion type and percentage of BSA percentage Skin biopsy (lichenoid dermatitis, spongiotic dermatitis, perivascular infiltrate rich in T lymphocytes, in bulbous dermatosessubepidermal blisters can be observed)	Inflammatory dermatitis, rush
	Vitiligo-like lesions, usually bilaterally and symmetrically distributed	Full skin and mucosal examination with attention to lesion type and percentage of BSA percentage Skin biopsy (lichenoid dermatitis, spongiotic dermatitis, perivascular infiltrate rich in T lymphocytes, in bulbous dermatoses subepidermal blisters can be observed)	Vitiligo
	Pemphigoid Skin blisters	Full skin and mucosal examination with attention to lesion type and percentage of BSA percentage Skin biopsy (lichenoid dermatitis, spongiotic dermatitis, perivascular infiltrate rich in T lymphocytes, in bulbous dermatoses subepidermal blisters can be observed)	Bullous dermatoses
	Changes in structure of skin Skin pain Fever Malaise Myalgias Arthralgias Abdominal pain Mucositis Lymphadenopathy	Nikolsky sign present (swelling andwrinkling with detachment of upperlayers of the skin)	Severe Cutaneous Adverse Reactions(SCARs): Steven-Johnson syndrome,toxic epidermal necrolysis (TEN), acute generalized exanthematous pustulosis, DRESS/DIHS

**Table 18:** Incidence of all-grade dermatological adverse events in cancer patients treated with ICI [66, 71, 74–79, 81, 130]

Drugs/irAE	Anti-PD-1/PD-L1	Anti-CTLA-4	Combined treatment
Dermatological toxicity	17–37% [79] 30–40% [77, 81] 34% [130] 37–42% [75]	37–70% [79] 44–68% [75] 45% [130] 50% [77, 81]	58–71% [75]
Rush	0.7–16.1% [74] 14.3–16.7% [71, 77] 17.1–19.2% [78] 25.9% [66]	19.1–34.2% [74] 20.5–31.1% [78] 24.3% [77] 32.8% [66]	16.7–30% [74] 39.8–41.8% [78] 40.3% [66]
Pruritus	18.8% [66]	35.4% [66] 24.9–26.8% [78]	33.2% [66]
Rush/pruritus	13–20% [81] 27.5–44.7% [76]	39.9–58.7% [76]	71.3% [76]
Vitiligo	8% [81]	NR	NR

*NR* not reported

Despite these being the most common side effects, skin lesions are usually of a minor intensity, and complications at the G3/4 degree occur in only about 3% of treated patients [134]. Treatment of severe complications requires dermatological consultation and sometimes also hospitalization.

Patients most often complain of a skin rash (24% with ipilimumab, 15% with anti-PD-1 and 40% with a combination of these two agents), pruritus (30% with ipilimumab, 15% with anti-PD-1, and about 33% with combination treatment), and vitiligo (about 8% with anti-PD-1, rarely with ipilimumab) [130]. Interestingly, one study showed a significant relationship between the occurrence of vitiligo and the clinical response to treatment. This symptom was much more common in patients treated with immunotherapy due to skin melanoma than in patients treated with other neoplasms, such as kidney or lung cancer [135]. A similar correlation with skin rash was also observed in later studies in patients treated with nivolumab in whom there was a good response to treatment with improved ORR and extended OS [136].

Other less frequently reported symptoms related to immunotherapy are photosensitivity, alopecia areata, xerosis cutis, or stomatitis. There have been reports of exacerbation of psoriasis during immunocompetent therapy [137].

In patients undergoing immunotherapy, who report skin-related concerns allegedly caused by treatment, it is essential, first of all, to exclude other possible causes, such as infection, the effect of other drugs, or the influence of other conditions, taking into account general symptoms (fever, lymphadenopathy), and if necessary, perform any necessary investigations. This is to eliminate potential dermatological emergencies such as Stevens-Johnson syndrome, toxic epidermal necrolysis, acute febrile neutrophilic dermatosis (Sweet syndrome), or drug rash with eosinophilia and systemic symptoms (DRESS). Immunotherapy is contraindicated in these conditions, and further treatment should be carried out under the supervision of a dermatologist or in a hospital environment. Treatment with high doses of steroids is required (e.g., methylprednisolone 2 mg/kg once or twice a day *i.v.*, with a gradual reduction of the dose over 4 weeks as improvement occurs). However, if skin lesions are diagnosed as G1 complications, treatment with antihistamines, local treatment of pruritus, continuation of immunotherapy, and the use of emollients are advised. In the case of complications at the G2 stage, topical glucocorticoids should be used, and if there is no improvement, oral prednisone at a dose of 1 mg/kg/day or an equivalent dose, with a gradual reduction of the dose over 4 weeks as improvement is achieved, is recommended. Immunotherapy may be continued. Where skin lesions cover more than 30% of the body surface area (BSA) and symptoms significantly affect daily functioning (G3), the recommended treatment is to use high doses of glucocorticosteroids *p.o.* (prednisolone 1 mg/kg) or *i.v.* (methylprednisolone 2 mg/kg daily for several days), followed by dose reduction over 4 weeks. Immunotherapy should be suspended until symptoms resolve, or their intensity is reduced to at least G1.

In summary, the occurrence of G3 irAEs, after achieving remission, allows for the continuation of immunotherapy, while non-G3 adverse events often require termination of treatment with checkpoint inhibitors. The occurrence of severe complications at G4 often requires specialist treatment in a dermatology department and leads to termination of immunotherapy.

## Recommendations for the management of adverse events of nervous system origin

Assessment of the incidence of neurological complications is made difficult by the presence of paraneoplastic syndromes in patients treated for lung cancer but also because in some studies, lymphocytic pituitary inflammation was included in this group of complications, and which, due to its influence on hormonal function, and treatment should preferably be included with endocrine complications. Nevertheless, the frequency of irAEs related to the nervous system is estimated at about 4% of patients treated with ipilimumab and 6% of patients treated with anti-PD-1/PD-L1 antibodies. In the case of a combination of these two drugs, the frequency of symptoms increases to approximately 12%. Neurological disorders resulting from treatment of cancer patients with ICIs are summarized in Table 19, and their frequency is depicted in Table 20.

**Table 19:** All-grade adverse events of nervous system origin in cancer patients treated with ICI [71, 74, 76, 77, 79–81]

System	Symptoms	Abnormalities in diagnostic test results	Suspected pathology
Nervous system	Bilateral motor/ sensory/bowel and bladder signs/symptoms-loss of bowel/bladder function Pain in the lower back, neck, arm, or leg Tingling, numbness, or weakness Difficulty walking Abnormal/increased reflexes in extremities Decreased fine motor skills, balance and coordination	Lumbar puncture for CSF analysis— cytology/flow cytometry of the cerebrospinal fluid (normal/lymphocytosis with elevated proline) Spine/brain MRI (including axial sections through the region of suspected abnormality)	Myelopathy
	Fatigable/fluctuating muscle weakness (more proximal) Ocular/bulbar involvement (ptosis, extra ocular movement) Double vision Dysphagia Dysarthria Facial muscle weakness neck/respiratory muscle weakness Myositis Myocarditis	Brain MRI (no leptomeningeal or cranial nerve enhancement, parenchymal alterations) EMG (pathological jitter)	Myasthenia gravis Myasthenia-like syndrome
	Acute polyneuropathy Symmetrical muscle weakness Sensory symptoms Neuropathic pain localized to lower back and thighs Dysregulation of autonomic nerves	Anti-ganglioside, anti-acetylcholine receptor, and anti-strained muscle antibodies can be present Lumbar puncture (elevated WBC)	Guillain-Barre syndrome
	Asymmetric/symmetric sensory, motor, sensory-motor deficit Focal mononeuropathies Numbness Paresthesia Hypo-/areflexia Sensory ataxia	Nerve biopsy (to distinguish from direct tumor infiltration) MRI (to evaluate cranial neuropathies/nerve root abnormality) EMG	Peripheral neuropathy
	Symptoms related to nerves involved (proximal/distal peripheral sensory and motor nerves, autonomic nervous system), e.g., arrhythmias, silent angina due to damage to nerve fibers and disruption of pain transmission, gastroparesis, severe constipation, bladder paralysis Sweating abnormalities Sluggish pupil reaction Orthostatic hypertension	Abnormal electrophysiological tests	Autonomic neuropathy (sensory-motor)
	Headache Photophobia Neck stiffness Nausea/vomiting	Lumbar puncture for CSF analysis—cytology/flow cytometry of the cerebrospinal fluid (WBC <500, normal glucose)	Aseptic meningitis
	Confusion Fatigue Spastic tremors Fever Vomiting Altered behavior Headache Seizures Short-term memory loss Lowered level of consciousness Focal weakness Speech abnormality Cerebral symptoms (gait disturbance, tremor, altered movements)	Lumbar puncture for CSF analysis—cytology/flow cytometry of the cerebrospinal fluid (WBC <250, mononuclear pleocytosis, normal glucose, increased protein level) Brain MRI (diffuse dural enhancement without parenchymal abnormalities) EEG (diffuse non-specific slowing) Anti-NMDA receptor antibodies positive in some cases	Encephalitis
	Acute/ subacute weakness Bilateral sensory changes Increased deep tendon reflex	MRI (inflammation of the spinal cord and other potential causes) Lumbar puncture (± abnormally high numbers of white blood cells or immune system proteins that indicate inflammation) Blood tests (± antibodies associated with neuromyelitis optica, a condition in which inflammation occurs both in the spinal cord and in the optic eye)	Transverse myelitis

**Table 20:** Incidence of all-grade neurological adverse events in cancer patients treated with ICI [74, 78–81]

Drugs/irAE	Anti-PD-1/PD-L1	Anti-CTLA-4	Combined treatment
Neurotoxicity	0.3–1% [74] 1% [80] 6% [81] 6.1% [79]	3.8% [79] <4% [81] 4.5% [74]	12.0% [79, 81]
Guillain-Barre syndrome	0.1–0.2% [78]	0.01% [78]	0–0.4% [78]
Myasthenia gravis, Myasthenia-like syndrome	NR	1.3% [78]	0–1.5% [78]
Neuropathy	NR	0.6% [78]	0–1.5% [78]

*NR* not reported

Adverse neurological symptoms most often appear between the 6th and 8th week of therapy and are relatively mild. In the main, they consist of headache, dizziness, or taste disturbances. Neurological disorders in the field of peripheral nervous system dysfunction occur rarely, but serious complications such as acute inflammatory demyelinating polyradiculoneuropathy (Guillain-Barre Syndrome), severe forms of myasthenia gravis, or peripheral polyneuropathy usually require treatment with long-term steroid therapy and in the case of resistance to such treatment, immunoglobulins, plasmapheresis, or immunosuppressants (e.g. azathioprine) may be used, which will usually require the involvement of a neurologist [138–140].

Serious neurological disorders have been observed with the use of vemurafenib after treatment with anti-PD-1 drugs [141].

## Recommendations for the management of adverse events affecting the vision system

Ocular complications are extremely rare and can be observed in fewer than 1% of patients. They may appear both in the first weeks of therapy and later [142]. Ocular complications include uveitis, episcleritis, iritis, and conjunctivitis. Uveitis is a serious complication, which manifests itself as visual impairment. In such cases, it is advisable to consult an ophthalmologist to initiate treatment and to discontinue immunotherapy (often permanent discontinuation). Topical preparations can be used in the case of local adverse events such as dryness. Ocular disorders resulting from treatment of cancer patients with ICIs are listed in Table 21, and their frequency is presented in Table 22. Patients previously treated with BRAF/MEK inhibitors, in whom an accumulation of adverse events may be observed, require special attention.

**Table 21:** All-grade ocular adverse events in cancer patients treated with ICI [71, 74, 76–81]

System	Organ	Symptoms	Abnormalities in diagnostic test results	Suspected pathology
Ocular	Middle layer of the eye	Blurred vision Floaters Flashing lights Eye redness Change in color vision Photophobia/ light sensitivity Visual distortion Scotomas Visual field changes Double vision Tenderness Pain with eye movement Eyelid swelling Proptosis Scotomas Tender eyes	Clinical examination (visual acuity, color vision, test for afferent pupillary defect) Ophthalmoscopy	Uveitis Iritis
	Episcleral tissue		Clinical examination (difference in redness of eye)	Episcleritis
	Eyelid		Clinical examination (the presence of scurf, telangiectatic vascular changes of the eyelid margin, inspissated meibomian glands, conjunctival hyperemia, punctuate keratopathy, cornea vascularization, and ulceration)	Blepharitis
	Uvea	Conjunctival redness Eye pain Photophobia Floaters Blurred vision	Ophthalmologic examination Funduscopic examination Fluorescein angiography Electrophysiological examination	Uveitis
		Eye signs (blurred vision, bilateral uveitis) Inner ear signs (hearing loss) Neurological signs (acute encephalitis signs, headache, meningismus) Cutaneous demonstration (vitiligo, alopecia)	Ocular coherence tomography (exudative detachments of the retina in the acute stage, along with choroidal thickening and demonstrating choroidal thinning in the chronic stage)	Vogt-Koyanagi-Harada syndrome (uveomeningitis)

**Table 22:** Incidence of all-grade ocular adverse events in cancer patients treated with ICI [76, 78, 80, 101]

Drugs/irAE	Anti-PD-1/PD-L1	Anti-CTLA-4	Combined treatment
Ocular toxicity	0–0.4% [76]	1% [80]	2.6% [78]
Blurred vision	1.5% [101]	NR	2.8% [101]
Uveitis	0.2–0.7% [78]	0.9% [78]	0–2.6% [78]

*NR* not reported

## Conclusions

The treatment of cancer patients with immune checkpoint inhibitors has undoubtedly been a significant breakthrough in the field of oncology in recent years. The possibility of blocking the PD-1/PD-L1 immune checkpoint provided an opportunity for achieving treatment results that could not have been envisaged with standard chemotherapy. The use of ICIs will steadily increase with the implementation of new indications, their administration at earlier stages of cancer (neoadjuvant, adjuvant treatment), and simply because such therapy will become more affordable. New avenues based on concepts utilizing yet unexploited anticancer treatments combining ICIs with targeted therapies, eg. antiangiogenic modalities have become available. With the expansion of the use of checkpoint inhibitors, it is also to be expected that doctors will be faced ever increasingly with having to manage the adverse events associated with these drugs. New treatment options pose new challenges not only for oncologists but also for specialists in other clinical fields, as well as for general practitioners. They also endorse the need for taking a holistic approach to the patient, a principle that is widely recognized in oncology. This is especially important because of the wide variety of organ complications that may affect patients treated with the expanding use of immunotherapy. It should be borne in mind that although serious and life-threatening complications are rare, patients will report systemic or organ symptoms of varying severity. The basis for any management procedure is to provide appropriate patient education and ensure multidisciplinary cooperation and adherence to diagnostic and therapeutic recommendations. Knowledge and awareness of the spectrum of adverse events accompanying immunotherapy will allow doctors to better qualify patients for treatment, prevent complications, correctly recognize, and ultimately treat them. Most of the general symptoms will be reported to general practitioners, as they may appear even after the termination of treatment and do not always proceed in line with disease progression. Specialists in various fields, e.g., endocrinologists, dermatologists, pulmonologists, and gastroenterologists, will often receive referrals for patients suffering these types of adverse events or will be asked to provide care in cases requiring hospitalization of patients with complications in their field of expertise. In view of these considerations, we believe that there is an urgency for multidisciplinary teams to work together in the treatment of cancer patients undergoing immunotherapy and suffering the consequent adverse events effects of treatment.

Therapeutic management should be adjusted according to the irAEs present. In the presence of G1 complications, immunotherapy may be continued under constant supervision. However, discontinuation of the use of checkpoint inhibitors is recommended in the case of neurological, hematological, and cardiac toxicity. For G2 level complications, treatment should be temporarily discontinued until symptoms resolve to G1 or toxicity resolves. In G3, the administration of immunotherapy should be permanently discontinued, and high doses of glucocorticosteroids are used in the therapy. G4 complications often require hospitalization and systemic treatment [79]. G5 complications are defined as fatal. Hence careful diagnostic procedures and early detection of the complications associated with treatment used are vitally important. As has already been stated, there are standards available for the management of adverse events of immune origin in the form of recommendations of the National Comprehensive Cancer Network (NCCN) in cooperation with American Society of Clinical Oncology (ASCO) [79], European Society for Medical Oncology (ESMO) [143], and the Society for Immunotherapy of Cancer (SITC) [81].

## Abbreviations

acquired TTP: acquired thrombotic thrombocytopenic purpura
ACTH: adrenocorticotropic hormone
ADCC: antibody-dependent cell-mediated cytotoxicity
ADL: activities of daily living
AIN: acute interstitial nephritis
AKI: acute kidney injury
AKT: protein kinase B
ANA: antinuclear antibodies
anti-CCP: anti-cyclic citrullinated peptides
anti-TPO: anti-thyroid peroxidase
APC: antigen-presenting cell
BAL: bronchoalveolar lavage
Bcl-2: B-cell lymphoma
Bcl-xL: B-cell extra-large lymphoma
BSA: body surface area
CDKS: cyclin-dependent kinases
CIP: checkpoint inhibitor pneumonitis
CK: creatine kinase
COPD: chronic obstructive pulmonary disease
CR: complete response
CSCC: cutaneous squamous cell carcinoma
CTCAE: Common Terminology Criteria for Adverse Events
CTLA-4: cytotoxic T cell antigen 4
DIPG: diffuse intrinsic pontine glioma
DLBCL: diffuse large B-cell lymphoma
DMARDs: disease-modifying drugs
DRESS: drug rash with eosinophilia and systemic symptoms
ESR: erythrocyte sedimentation rate
FL: follicular lymphoma
fT4: free thyroxine
GPs: general practitioners
HCC: hepatocellular carcinoma
HNSCC: head and neck squamous cell cancer
HUS: Hemolytic uremic syndrome
ICIs: immune checkpoint inhibitors
ICOS: inducible T-cell costimulator (CD 278)
ICU: intensive care unit
IDD: diabetes type 1, insulin-dependent diabetes mellitus
ITP: immune thrombocytes purpura
IFN-γ: interferon γ
IL-2: interleukin 2
irAE: immunotherapy-related adverse events
MHC: major histocompatibility complex
MCC: Merkel cell carcinoma
MMF: mycophenolate mofetil
MSI: microsatellite instability
MMR: mismatch repair
mTOR: mammalian target of rapamycin
NCI: National Cancer Institute’s
NFAT: nuclear factor of activated T-cell
NF-KB: nuclear factor kB
NSCLC: non-small cell lung cancer
ORR: objective response rate
OS: overall survival
p27: protein regulating cell cycle
PAI: primary adrenal insufficiency
PD-1: programmed cell death protein 1
PD-L1: programmed death-ligand 1
PFS: progression free survival
PFTs: pulmonary function tests
PI 3K: phosphatidylinositol 3-kinase
PLCY: phospholipase C gamma
PP2A: protein phosphate 2A
RF: rheumatoid factor
Shp2: protein tyrosine phosphate 2
SKP2: S-phase kinase-associated protein 2
T3: triiodothyronine
TCR: T-cell receptor
TEN: toxic epidermal necrolysis
TRAb: thyroid stimulating hormone receptor antibody
TSH: thyroid stimulating hormone

## References

[1] Larkin J, Chiarion-Sileni V, Gonzalez R, Grob JJ, Cowey CL, Lao CD, Wagstaff J, Hogg D, Hill A, Carlino MS, Wolter P, Lebbé C, Schachter J, Thomas L, Hassel JC, Lorigan P, Walker D, Jiang J, Hodi FS, Wolchok JD (2015). Efficacy and safety in key patient subgroups of nivolumab (NIVO) alone or combined with ipilimumab (IPI) versus IPI alone in treatment-naıve patients with advanced melanoma (MEL) (CheckMate 067). European Journal of Cancer.

[2] Swann JB, Smyth MJ (2007). Immune surveillance of tumors. Journal of Clinical Investigation.

[3] Xin Yu J, Hubbard-Lucey VM, Tang J (2019). Immuno-oncology drug development goes global. Nature Reviews Drug Discovery.

[4] Lenschow DJ, Walunas TL, Bluestone JA (1996). CD28/B7 system of T cell costimulation. Annual Review of Immunology.

[5] Ishida Y, Agata Y, Shibahara K, Honjo T (1992). Induced expression of PD-1, a novel member of the immunoglobulin gene superfamily, upon programmed cell death. The EMBO Journal.

[6] Keir, M. E., Butte, M. J, Freeman, G. J., & Sharpe, A.H. (2008). PD-1 and its ligands in tolerance and immunity. *Annual Review of Immunology*, 26, 677–704. 10.1146/annurev.immunol.26.021607.090331 10.1146/annurev.immunol.26.021607.090331 PMC10637733 18173375

[7] Yamazaki T, Akiba H, Iwai H, Matsuda H, Aoki M, Tanno Y, Shin T, Tsuchiya H, Pardoll DM, Okumura K, Azuma M, Yagita H (2002). Expression of programmed death 1 ligands by murine T cells and APC. Journal of Immunology.

[8] Dong H, Zhu G, Tamada K, Chen L (1999). B7-H1, a third member of the B7 family, costimulates T-cell proliferation and interleukin-10 secretion. Nature Medicine.

[9] Latchman Y, Wood CR, Chernova T, Chaudhary D, Borde M, Chernova I, Iwai Y, Long AJ, Brown JA, Nunes R, Greenfield EA, Bourque K, Boussiotis VA, Carter LL, Carreno BM, Malenkovich N, Nishimura H, Okazaki T, Honjo T, Sharpe AH, Freeman GJ (2001). PD-L2 is a second ligand for PD-1 and inhibits T cell activation. Nature Immunology.

[10] Brahmer JR, Drake CG, Wollner I, Powderly JD, Picus J, Sharfman WH, Stankevich E, Pons A, Salay TM, McMiller TL, Gilson MM, Wang C, Selby M, Taube JM, Anders R, Chen L, Korman AJ, Pardoll DM, Lowy I, Topalian SL (2010). Phase I study of single-agent anti-programmed death-1 (MDX-1106) in refractory solid tumors: Safety, clinical activity, pharmacodynamics, and immunologic correlates. Journal of Clinical Oncology.

[11] Patsoukis N, Brown J, Petkova V, Liu F, Li L, Boussiotis VA (2012). Selective effects of PD-1 on Akt and Ras pathways regulate molecular components of the cell cycle and inhibit T cell proliferation. Science Signaling.

[12] Thompson, R. H., Gillett, M. D., Cheville, J. C., Lohse, C. M., Dong, H., Webster, W. S., Krejci, K. G., Lobo, J. R., Sengupta, S., Chen, L., Zincke, H., Blute, M. L., Strome, S. E., Leibovich, B. C., & Kwon, E. D. (2004). Costimulatory B7-H1 in renal cell carcinoma patients: Indicator of tumor aggressiveness and potential therapeutic target. *Proceedings of the National Academy of Sciences of the United States of America, 101*, 17174–17179. 10.1073/pnas.0406351101. 10.1073/pnas.0406351101 PMC534606 15569934

[13] Hino, R., Kabashima, K., Kato, Y., Yagi, H., Nakamura, M., Honjo, T., Okazaki, T., & Tokura, Y. (2010). Tumor cell expression of programmed cell death-1 ligand 1 is a prognostic factor for malignant melanoma. *Cancer, 116*(7), 1757–1766. 10.1002/cncr.24899. 10.1002/cncr.24899 20143437

[14] Zeng, Z., Shi, F., Zhou, L., Zhang, M. N., Chen, Y., Chang, X. J., Lu, Y. Y., Bai, W. L., Qu, J. H., Wang, C. P., Wang, H., Lou, M., Wang, F. S., Lv, J. Y., & Yang, Y. P. (2011). Upregulation of circulating PD-L1/PD-1 is associated with poor post-cryoablation prognosis in patients with HBV-related hepatocellular carcinoma. *PLoS One, 6*(9), e23621. 10.1371/journal.pone.0023621. 10.1371/journal.pone.0023621 PMC3164659 21912640

[15] Gao, Q., Wang, X. Y., Qiu, S. J., Yamato, I., Sho, M., Nakajima, Y., Zhou, J., Li, B. Z., Shi, Y. H., Xiao, Y. S., Xu, Y., & Fan, J. (2009). Overexpression of PD-L1 significantly associates with tumor aggressiveness and postoperative recurrence in human hepatocellular carcinoma. *Clinical Cancer Research, 15*, 971–979. 10.1158/1078-0432.ccr-08-1608. 10.1158/1078-0432.CCR-08-1608 19188168

[16] Mu, C. Y., Huang, J. A., Chen, Y., Chen, C., & Zhang, X. G. (2011). High expression of PD-L1 in lung cancer may contribute to poor prognosis and tumor cells immune escape through suppressing tumor infiltrating dendritic cells maturation. *Medical Oncology, 28*, 682–688. 10.1007/s12032-010-9515-2. 10.1007/s12032-010-9515-2 20373055

[17] Zhang, Y., Huang, S., Gong, D., Qin, Y., & Shen, Q. (2010). Programmed death-1 upregulation is correlated with dysfunction of tumor-infiltrating CD8+ T lymphocytes in human non-small cell lung cancer. *Cellular & Molecular Immunology, 7*, 389–395. 10.1038/cmi.2010.28. 10.1038/cmi.2010.28 PMC4002677 20514052

[18] Taube JM, Anders RA, Young GD, Xu H, Sharma R, McMiller TL, Chen S, Klein AP, Pardoll DM, Topalian SL, Chen L (2012). Colocalization of inflammatory response with B7-h1 expression in human melanocytic lesions supports an adaptive resistance mechanism of immune escape. Science Translational Medicine.

[19] Zhou Q, Xiao H, Liu Y, Peng Y, Hong Y, Yagita H, Chandler P, Munn DH, Mellor A, Fu N, He Y (2010). Blockade of programmed death-1 pathway rescues the effector function of tumor-infiltrating T cells and enhances the antitumor efficacy of lentivector immunization. Journal of Immunology.

[20] Nomi T, Sho M, Akahori T, Hamada K, Kubo A, Kanehiro H, Nakamura S, Enomoto K, Yagita H, Azuma M, Nakajima Y (2007). Clinical significance and therapeutic potential of the programmed death-1 ligand/programmed death-1 pathway in human pancreatic cancer. Clinical Cancer Research.

[21] Iwai Y, Terawaki S, Honjo T (2005). PD-1 blockade inhibits hematogenous spread of poorly immunogenic tumor cells by enhanced recruitment of effector T cells. International Immunology.

[22] Curran MA, Montalvo W, Yagita H, Allison JP (2010). PD-1 and CTLA-4 combination blockade expands infiltrating T cells and reduces regulatory T and myeloid cells within B16 melanoma tumors. Proceedings of the National Academy of Sciences of the United States of America.

[23] Larkin J, Minor D, D’Angelo S, Neyns B, Smylie M, Miller WH (2018). Overall survival in patients with advanced melanoma who received nivolumab versus investigator’s choice chemotherapy in CheckMate 037: a randomized, controlled, open-label phase III trial. Journal of Clinical Oncology, 1.

[24] Wolchok JD, Chiarion-Sileni V, Gonzalez R, Rutkowski P, Grob JJ, Cowey CL, Lao CD, Wagstaff J, Schadendorf D, Ferrucci PF, Smylie M, Dummer R, Hill A, Hogg D, Haanen J, Carlino MS, Bechter O, Maio M, Marquez-Rodas I, Guidoboni M, McArthur G, Lebbé C, Ascierto PA, Long GV, Cebon J, Sosman J, Postow MA, Callahan MK, Walker D, Rollin L, Bhore R, Hodi FS, Larkin J (2017). Overall survival with combined nivolumab and ipilimumab in advanced melanoma. The New England Journal of Medicine.

[25] Weber J, Mandala M, Del Vecchio M, Gogas HJ, Gogas HJ, Arance AM (2017). Adjuvant nivolumab versus ipilimumab in resected stage III or IV melanoma. The New England Journal of Medicine.

[26] Brahmer J, Reckamp KL, Baas P, Crinò L, Eberhardt WE, Poddubskaya E (2015). Nivolumab versus docetaxel in advanced squamous-cell non–small-cell lung cancer. The New England Journal of Medicine.

[27] Borghaei H, Paz-Ares L, Horn L, Spigel DR, Steins M, Ready NE, Chow LQ, Vokes EE, Felip E, Holgado E, Barlesi F, Kohlhäufl M, Arrieta O, Burgio MA, Fayette J, Lena H, Poddubskaya E, Gerber DE, Gettinger SN, Rudin CM, Rizvi N, Crinò L, Blumenschein GR, Antonia SJ, Dorange C, Harbison CT, Graf Finckenstein F, Brahmer JR (2015). Nivolumab versus docetaxel in advanced nonsquamous non-small-cell lung cancer. The New England Journal of Medicine.

[28] Ferris RL, Blumenschein G, Fayette J, Guigay J, Colevas AD, Licitra L, Harrington K, Kasper S, Vokes EE, Even C, Worden F, Saba NF, Iglesias Docampo LC, Haddad R, Rordorf T, Kiyota N, Tahara M, Monga M, Lynch M, Geese WJ, Kopit J, Shaw JW, Gillison ML (2016). Nivolumab for recurrent squamous-cell carcinoma of the head and neck. The New England Journal of Medicine.

[29] Sharma P, Retz M, Siefker-Radtke A, Baron A, Necchi A, Bedke J, Plimack ER, Vaena D, Grimm MO, Bracarda S, Arranz JÁ, Pal S, Ohyama C, Saci A, Qu X, Lambert A, Krishnan S, Azrilevich A, Galsky MD (2017). Nivolumab in metastatic urothelial carcinoma after platinum therapy (CheckMate 275): A multicentre, single-arm, phase 2 trial. The Lancet Oncology.

[30] Motzer RJ, Escudier B, McDermott DF, George S, Hammers HJ, Srinivas S (2015). Nivolumab versus everolimus in advanced renal-cell carcinoma. The New England Journal of Medicine.

[31] El-Khoueiry AB, Sangro B, Yau T, Crocenzi TS, Kudo M, Hsu C (2017). Nivolumab in patients with advanced hepatocellular carcinoma (CheckMate 040): An open-label, non-comparative, phase 1/2 dose escalation and expansion trial. Lancet.

[32] Overman MJ, McDermott R, Leach JL, Lonardi S, Lenz HJ, Morse MA, Desai J, Hill A, Axelson M, Moss RA, Goldberg MV, Cao ZA, Ledeine JM, Maglinte GA, Kopetz S, André T (2017). Nivolumab in patients with metastatic DNA mismatch repair-deficient or microsatellite instability-high colorectal cancer (CheckMate 142): An open-label, multicentre, phase 2 study. The Lancet Oncology.

[33] Hamanishi J, Mandai M, Ikeda T, Minami M, Kawaguchi A, Murayama T, Kanai M, Mori Y, Matsumoto S, Chikuma S, Matsumura N, Abiko K, Baba T, Yamaguchi K, Ueda A, Hosoe Y, Morita S, Yokode M, Shimizu A, Honjo T, Konishi I (2015). Safety and antitumor activity of anti–PD-1 antibody, nivolumab, in patients with platinum-resistant ovarian cancer. Journal of Clinical Oncology.

[34] Ansell SM, Lesokhin AM, Borrello I, Halwani A, Scott EC, Gutierrez M, Schuster SJ, Millenson MM, Cattry D, Freeman GJ, Rodig SJ, Chapuy B, Ligon AH, Zhu L, Grosso JF, Kim SY, Timmerman JM, Shipp MA, Armand P (2015). PD-1 blockade with nivolumab in relapsed or refractory Hodgkin’s lymphoma. The New England Journal of Medicine.

[35] Robert C, Schachter J, Long GV, Arance A, Grob JJ, Mortier L, Daud A, Carlino MS, McNeil C, Lotem M, Larkin J, Lorigan P, Neyns B, Blank CU, Hamid O, Mateus C, Shapira-Frommer R, Kosh M, Zhou H, Ibrahim N, Ebbinghaus S, Ribas A (2015). Pembrolizumab versus ipilimumab in advanced melanoma. The New England Journal of Medicine.

[36] Ribas A, Puzanov I, Dummer R, Schadendorf D, Hamid O, Robert C, Hodi FS, Schachter J, Pavlick AC, Lewis KD, Cranmer LD, Blank CU, O'Day SJ, Ascierto PA, Salama AKS, Margolin KA, Loquai C, Eigentler TK, Gangadhar TC, Carlino MS, Agarwala SS, Moschos SJ, Sosman JA, Goldinger SM, Shapira-Frommer R, Gonzalez R, Kirkwood JM, Wolchok JD, Eggermont A, Li XN, Zhou W, Zernhelt AM, Lis J, Ebbinghaus S, Kang SP, Daud A (2015). Pembrolizumab versus investigator-choice chemotherapy for ipilimumab-refractory melanoma (KEYNOTE-002): A randomised, controlled, phase 2 trial. The Lancet Oncology.

[37] Reck M, Rodríguez-Abreu D, Robinson AG, Hui R, Csőszi T, Fülöp A, Gottfried M, Peled N, Tafreshi A, Cuffe S, O’Brien M, Rao S, Hotta K, Leiby MA, Lubiniecki GM, Shentu Y, Rangwala R, Brahmer JR (2016). Pembrolizumab versus chemotherapy for PD-L1-positive non-small-cell lung cancer. The New England Journal of Medicine.

[38] Gandhi L, Rodríguez-Abreu D, Gadgeel S, Esteban E, Felip E, De Angelis F (2018). Pembrolizumab plus chemotherapy in metastatic non-small-cell lung cancer. The New England Journal of Medicine.

[39] Bellmunt J, de Wit R, Vaughn DJ, Fradet Y, Lee JL, Fong L, Vogelzang NJ, Climent MA, Petrylak DP, Choueiri TK, Necchi A, Gerritsen W, Gurney H, Quinn DI, Culine S, Sternberg CN, Mai Y, Poehlein CH, Perini RF, Bajorin DF, KEYNOTE-045 Investigators (2017). Pembrolizumab as second-line therapy for advanced urothelial carcinoma. New England Journal of Medicine.

[40] Balar AV, Castellano D, O’Donnell PH, Grivas P, Vuky J, Powles T (2017). First-line pembrolizumab in cisplatin-ineligible patients with locally advanced and unresectable or metastatic urothelial cancer (KEYNOTE-052): a multicentre, single-arm, phase 2 study. The Lancet Oncology.

[41] Westin JR, Chu F, Zhang M, Fayad LE, Kwak LW, Fowler N, Romaguera J, Hagemeister F, Fanale M, Samaniego F, Feng L, Baladandayuthapani V, Wang Z, Ma W, Gao Y, Wallace M, Vence LM, Radvanyi L, Muzzafar T, Rotem-Yehudar R, Davis RE, Neelapu SS (2014). Safety and activity of PD1 blockade by pidilizumab in combination with rituximab in patients with relapsed follicular lymphoma: A single group, open-label, phase 2 trial. The Lancet Oncology.

[42] Armand P, Nagler A, Weller EA, Devine SM, Avigan DE, Chen YB, Kaminski MS, Holland HK, Winter JN, Mason JR, Fay JW, Rizzieri DA, Hosing CM, Ball ED, Uberti JP, Lazarus HM, Mapara MY, Gregory SA, Timmerman JM, Andorsky D, Or R, Waller EK, Rotem-Yehudar R, Gordon LI (2013). Disabling immune tolerance by programmed death-1 blockade with pidilizumab after autologous hematopoietic stem-cell transplantation for diffuse large B-cell lymphoma: Results of an international phase II trial. Journal of Clinical Oncology.

[43] Camicia R, Winkler HC, Hassa PO (2015). Novel drug targets for personalized precision medicine in relapsed/refractory diffuse large B-cell lymphoma: A comprehensive review. Molecular Cancer.

[44] Mahoney KM, Freeman GJ, McDermott DF (2015). The next immune-checkpoint inhibitors: PD-1/PD-L1 blockade in melanoma. Clinical Therapeutics.

[45] Fried I, Lossos A, Ben Ami T, Dvir R, Toledano H, Ben Arush MW, Postovski S, Abu Kuidar A, Yalon M, Weintraub M, Benifla M (2018). Preliminary results of immune modulating antibody MDV9300 (pidilizumab) treatment in children with diffuse intrinsic pontine glioma. Journal of Neuro-Oncology.

[46] Rischin D, Migden MR, Lim AM, Schmults CD, Khushalani NI, Hughes BGM, Schadendorf D, Dunn LA, Hernandez-Aya L, Chang ALS, Modi B, Hauschild A, Ulrich C, Eigentler T, Stein B, Pavlick AC, Geiger JL, Gutzmer R, Alam M, Okoye E, Mathias M, Jankovic V, Stankevich E, Booth J, Li S, Lowy I, Fury MG, Guminski A (2020). Phase 2 study of cemiplimab in patients with metastatic cutaneous squamous cell carcinoma: Primary analysis of fixed-dosing, long-term outcome of weight-based dosing. Journal for Immunotherapy of Cancer.

[47] Migden MR, Rischin D, Schmults CD, Guminski A, Hauschild A, Lewis KD, Chung CH, Hernandez-Aya L, Lim AM, Chang ALS, Rabinowits G, Thai AA, Dunn LA, Hughes BGM, Khushalani NI, Modi B, Schadendorf D, Gao B, Seebach F, Li S, Li J, Mathias M, Booth J, Mohan K, Stankevich E, Babiker HM, Brana I, Gil-Martin M, Homsi J, Johnson ML, Moreno V, Niu J, Owonikoko TK, Papadopoulos KP, Yancopoulos GD, Lowy I, Fury MG (2018). PD-1 Blockade with cemiplimab in advanced cutaneous squamous-cell carcinoma. The New England Journal of Medicine.

[48] Lee A, Duggan S, Deeks ED (2020). Cemiplimab: A review in advanced cutaneous squamous cell carcinoma. Adis Drug Reviews.

[49] Balar AV, Galsky MD, Rosenberg JE, Powles T, Petrylak DP, Bellmunt J, Loriot Y, Necchi A, Hoffman-Censits J, Perez-Gracia JL, Dawson NA, van der Heijden M, Dreicer R, Srinivas S, Retz MM, Joseph RW, Drakaki A, Vaishampayan UN, Sridhar SS, Quinn DI, Durán I, Shaffer DR, Eigl BJ, Grivas PD, Yu EY, Li S, Kadel EE III, Boyd Z, Bourgon R, Hegde PS, Mariathasan S, Thåström A, Abidoye OO, Fine GD, Bajorin DF, IMvigor210 Study Group (2017). Atezolizumab as first-line treatment in cisplatin-ineligible patients with locally advanced and metastatic urothelial carcinoma: A single-arm, multicentre, phase 2 trial. Lancet.

[50] Rittmeyer A, Barlesi F, Waterkamp D, Park K, Ciardiello F, von Pawel J, Gadgeel SM, Hida T, Kowalski DM, Dols MC, Cortinovis DL, Leach J, Polikoff J, Barrios C, Kabbinavar F, Frontera OA, de Marinis F, Turna H, Lee JS, Ballinger M, Kowanetz M, He P, Chen DS, Sandler A, Gandara DR, OAK Study Group (2016). Atezolizumab versus docetaxel in patients with previously treated non-small-cell lung cancer (OAK): A phase 3, open-label, multicentre randomised controlled trial. Lancet.

[51] Antonia SJ, Villegas A, Daniel D, Vicente D, Murakami S, Hui R, Kurata T, Chiappori A, Lee KH, de Wit M, Cho BC, Bourhaba M, Quantin X, Tokito T, Mekhail T, Planchard D, Kim YC, Karapetis CS, Hiret S, Ostoros G, Kubota K, Gray JE, Paz-Ares L, de Castro Carpeño J, Faivre-Finn C, Reck M, Vansteenkiste J, Spigel DR, Wadsworth C, Melillo G, Taboada M, Dennis PA, Özgüroğlu M (2018). Overall survival with durvalumab after chemoradiotherapy in stage III NSCLC. New England Journal of Medicine.

[52] Powles T, O’Donnell PH, Massard C, Arkenau HT, Friedlander TW, Hoimes CJ (2017). Efficacy and safety of durvalumab in locally advanced or metastatic urothelial carcinoma: Updated results from a phase 1/2 open-label Study. JAMA Oncology.

[53] Boyerinas B, Jochems C, Fantini M, Heery CR, Gulley JL, Tsang KY, Schlom J (2015). Antibody-dependent cellular cytotoxicity activity of a novel anti-pd-l1 antibody avelumab (MSB0010718C) on Human Tumor Cells. Cancer Immunology Research.

[54] Collins JM, Gulley JL (2019). Product review: avelumab, an anti-PD-L1 antibody. Human Vaccines & Immunotherapeutics.

[55] D’Angelo SP, Russell J, Lebbé C, Chmielowski B, Gambichler T, Grob JJ, Kiecker F, Rabinowits G, Terheyden P, Zwiener I, Bajars M, Hennessy M, Kaufman HL (2018). Efficacy and safety of first-line avelumab treatment in patients with stage IV metastatic merkel cell carcinoma: A preplanned interim analysis of a clinical trial. JAMA Oncology.

[56] Larroquette M, Gross-Goupil M, Daste A, Robert G, Ravaud A, Domblides C (2019). Which place for avelumab in the management of urothelial carcinoma?. Expert Opinion on Biological Therapy.

[57] Powles T, Park SH, Voog E, Caserta C, Valderrama BP, Gurney H, Kalofonos H, Radulović S, Demey W, Ullén A, Loriot Y, Sridhar SS, Tsuchiya N, Kopyltsov E, Sternberg CN, Bellmunt J, Aragon-Ching JB, Petrylak DP, Laliberte R, Wang J, Huang B, Davis C, Fowst C, Costa N, Blake-Haskins JA, di Pietro A, Grivas P (2020). Avelumab maintenance therapy for advanced or metastatic urothelial carcinoma. The New England Journal of Medicine.

[58] Motzer RJ, Penkov K, Haanen J, Rini B, Albiges L, Campbell MT, Venugopal B, Kollmannsberger C, Negrier S, Uemura M, Lee JL, Vasiliev A, Miller WH, Gurney H, Schmidinger M, Larkin J, Atkins MB, Bedke J, Alekseev B, Wang J, Mariani M, Robbins PB, Chudnovsky A, Fowst C, Hariharan S, Huang B, di Pietro A, Choueiri TK (2019). Avelumab plus axitinib versus sunitinib for advanced renal-cell carcinoma. The New England Journal of Medicine.

[59] Wei SC, Duffy CR, Allison JP (2018). Fundamental mechanisms of immune checkpoint blockade therapy. Cancer Discovery.

[60] Zhao Y, Yang W, Huang Y, Cui R, Li X, Li B (2018). Evolving roles for targeting CTLA-4 in cancer immunotherapy. Cellular Physiology and Biochemistry.

[61] Rowshanravan B, Halliday N, Sansom DM (2018). CTLA-4: A moving target in immunotherapy. Blood.

[62] Schadendorf D, Hodi FS, Robert C, Weber JS, Margolin K, Hamid O, Patt D, Chen TT, Berman DM, Wolchok JD (2015). Pooled analysis of long-term survival data from phase II and phase III trials of ipilimumab in unresectable or metastatic melanoma. Journal of Clinical Oncology.

[63] Ribas A, Kefford R, Marshall MA, Punt CJ, Haanen JB, Marmol M (2013). Phase III randomized clinical trial comparing tremelimumab with standard-of-care chemotherapy in patients with advanced melanoma. Journal of Clinical Oncology.

[64] Cheng H, Sun G, Chen H, Li Y, Han Z, Li Y, Zhang P, Yang L, Li Y (2019). Trends in the treatment of advanced hepatocellular carcinoma: Immune checkpoint blockade immunotherapy and related combination therapies. American Journal of Cancer Research.

[65] Wiater K, Switaj T, Mackiewicz J, Kalinka-Warzocha E, Wojtukiewicz M, Szambora P (2013). Efficacy and safety of ipilimumab therapy in patients with metastatic melanoma: A retrospective multicenter analysis. Contemporary Oncology (Pozn).

[66] Larkin J, Chiarion-Sileni V, Gonzalez R, Jacques Grob J, Cowey L, Lao CD (2015). Combined nivolumab and ipilimumab or monotherapy in untreated melanoma. The New England Journal of Medicine.

[67] Lugowska I, Teterycz P, Rutkowski P (2018). Immunotherapy of melanoma. Contemporary Oncology.

[68] Hodi FS, Chesney J, Pavlick AC, Robert C, Grossmann KF, McDermott DF, Linette GP, Meyer N, Giguere JK, Agarwala SS, Shaheen M, Ernstoff MS, Minor DR, Salama AK, Taylor MH, Ott PA, Horak C, Gagnier P, Jiang J, Wolchok JD, Postow MA (2016). Combined nivolumab and ipilimumab versus ipilimumab alone in patients with advanced melanoma: 2-year overall survival outcomes in a multicentre, randomised, controlled, phase 2 trial. The Lancet Oncology.

[69] Weber, J. S. (2018). *Challenging cases: Management of immune-related toxicity* (Vol. 38, pp. 179–183). American Society of Clinical Oncology Educational Book. 10.1200/EDBK_209557 30231403

[70] Postow, M. A., Sidlow, R., & Hellmann, M. D. (2018). Immune-related adverse events associated with immune checkpoint blockade. *The New England Journal of Medicine, 378*(2), 158–168. 10.1056/nejmra1703481. 10.1056/NEJMra1703481 29320654

[71] Kennedy LB, Salama AKS (2020). A review of cancer immunotherapy toxicity. A Cancer Journal for Clinicians.

[72] https://ctep.cancer.gov/protocoldevelopment/electronic_applications/docs/ctcae_v5_quick_reference_5x7.pdf

[73] Borghaei H, Paz-Ares L, Horn L, Spigel DR, Steins M, Ready NE, Chow LQ, Vokes EE, Felip E, Holgado E, Barlesi F, Kohlhäufl M, Arrieta O, Burgio MA, Fayette J, Lena H, Poddubskaya E, Gerber DE, Gettinger SN, Rudin CM, Rizvi N, Crinò L, Blumenschein GR, Antonia SJ, Dorange C, Harbison CT, Graf Finckenstein F, Brahmer JR (2015). Nivolumab versus docetaxel in advanced nonsquamous non-small-cell lung cancer. The New England Journal of Medicine.

[74] Martins F, Sofiya L, Sykiotis GP, Lamine F, Maillard M, Fraga M, Shabafrouz K, Ribi C, Cairoli A, Guex-Crosier Y, Kuntzer T, Michielin O, Peters S, Coukos G, Spertini F, Thompson JA, Obeid M (2019). Adverse effects of immune-checkpoint inhibitors: Epidemiology, management and surveillance. Nature Reviews. Clinical Oncology.

[75] Darnell EP, Mooradian MJ, Baruch EN, Yilmaz M, Reynolds KL (2020). Immune-related adverse events (irAEs): Diagnosis, management, and clinical pearls. Current Oncology Reports.

[76] Kottschade L, Brys A, Peikert T, Ryder M, Raffals L, Brewer J, Mosca P, Markovic S, Midwest Melanoma Partnership (2016). Midwest Melanoma Partnership. A multidisciplinary approach to toxicity management of modern immune checkpoint inhibitors in cancer therapy. Melanoma Research.

[77] Domagała-Kulawik J, Leszek P, Owczarek W, Rawa T, Stelmachowska-Banaś M, Rutkowski P (2020). Immunotherapy of solid tumors: safety of treatment. *Polish*. Archives of Internal Medicine.

[78] Almutairi AR, McBride A, Slack M, Erstad BL, Abraham I (2020). Potential immune-related adverse events associated with monotherapy and combination therapy of ipilimumab, nivolumab, and pembrolizumab for advanced melanoma: A systematic review and meta-analysis. Frontiers in Oncology.

[79] Brahmer, J. R., Lacchetti, C., Schneider, B. J., Atkins, M. B., Brassil, K. J., Caterino, J. M., Chau, I., Ernstoff, M. S., Gardner, J. M., Ginex, P., Hallmeyer, S., Holter Chakrabarty, J., Leighl, N. B., Mammen, J. S., McDermott, D. F., Naing, A., Nastoupil, L. J., Phillips, T., Porter, L. D., Puzanov, I., Reichner, C. A., Santomasso, B. D., Seigel, C., Spira, A., Suarez-Almazor, M. E., Wang, Y., Weber, J. S., Wolchok, J. D., Thompson, J. A., & in collaboration with the National Comprehensive Cancer Network. (2018). Management of immune-related adverse events in patients treated with immune checkpoint inhibitor therapy: American Society of Clinical Oncology Clinical Practice Guideline. *Journal of Clinical Oncology, 17*, 1714–1768. 10.1200/jco.2017.77.6385. 10.1200/JCO.2017.77.6385 PMC6481621 29442540

[80] Baraibar, I., Melero, I., Ponz-Sarvise, M., & Castanon, E. (2019). Safety and tolerability of immune checkpoint inhibitors (PD-1 and PD-L1) in cancer. *Drug Safety, 42*(2), 281–294. 10.1007/s40264-018-0774-8. 10.1007/s40264-018-0774-8 30649742

[81] Puzanov, I., Diab, A., Abdallah, K., Bingham 3rd, C. O., Brogdon, C., Dadu, R., et al. (2017). Managing toxicities associated with immune checkpoint inhibitors: consensus recommendations from the Society for Immunotherapy of Cancer (SITC) Toxicity Management Working Group. *Journal for Immunotherapy of Cancer, 5*(1), 95. 10.1186/s40425-017-0300-z. 10.1186/s40425-017-0300-z PMC5697162 29162153

[82] Ueda H, Howson JM, Esposito L, Heward J, Snook H, Chamberlain G (2003). Association of the T-cell regulatory gene CTLA4 with susceptibility to autoimmune disease. Nature.

[83] Ferrari SM, Fallahi P, Galetta F, Citi E, Benvenga S, Antonelli A (2018). Thyroid disorders induced by checkpoint inhibitors. Reviews in Endocrine & Metabolic Disorders.

[84] Barroso-Sousa R, Barry WT, Garrido-Castro AC, Hodi FS, Min L, Krop IE, Tolaney SM (2018). Incidence of endocrine dysfunction following the use of different immune checkpoint inhibitor regimens: A systematic review and meta-analysis. JAMA Oncology.

[85] Corsello SM, Barnabei A, Marchetti P, De Vecchis L, Salvatori R, Torino F (2013). Endocrine side effects induced by immune checkpoint inhibitors. The Journal of Clinical Endocrinology and Metabolism.

[86] Byun DJ, Wolchok JD, Rosenberg LM, Girotra M (2017). Cancer immunotherapy - immune checkpoint blockade and associated endocrinopathies. Nature Reviews Endocrinology.

[87] Dillard T, Yedinak CG, Alumkal J, Fleseriu M (2010). Anti-CTLA-4 antibody therapy associated autoimmune hypophysitis: serious immune related adverse events across a spectrum of cancer subtypes. Pituitary.

[88] Sarnaik AA, Yu B, Yu D, Morelli D, Hall M, Bogle D, Yan L, Targan S, Solomon J, Nichol G, Yellin M, Weber JS (2011). Extended dose ipilimumab with a peptide vaccine: immune correlates associated with clinical benefit in patients with resected high-risk stage IIIc/IV melanoma. Clinical Cancer Research.

[89] Okamoto M, Okamoto M, Gotoh K, Masaki T, Ozeki Y, Ando H, Anai M, Sato A, Yoshida Y, Ueda S, Kakuma T, Shibata H (2016). Fulminant type 1 diabetes mellitus with anti-programmed cell death-1 therapy. Journal of Diabetes Investigation.

[90] de Filette JMK, Pen JJ, Decoster L, Vissers T, Bravenboer B, Van der Auwera BJ (2019). Immune checkpoint inhibitors and type 1 diabetes mellitus: A case report and systematic review. European Journal of Endocrinology.

[91] Weber JS, Dummer R, de Pril V, Lebbé C, Hodi FS, for the MDX010-20 Investigators (2013). Patterns of onset and resolution of immune-related adverse events of special interest with ipilimumab: detailed safety analysis from a phase 3 trial in patients with advanced melanoma. Cancer.

[92] Gupta, A., De Felice, K. M., Loftus Jr., E. V., & Khanna, S. (2015). Systematic review: Colitis associated with anti-CTLA-4 therapy. *Alimentary Pharmacology & Therapeutics, 42*, 406–417. 10.1111/apt.13281. 10.1111/apt.13281 26079306

[93] Horvat TZ, Adel NG, Dang TO, Momtaz P, Postow MA, Callahan MK, Carvajal RD, Dickson MA, D'Angelo SP, Woo KM, Panageas KS, Wolchok JD, Chapman PB (2015). Immune-related adverse events, need for systemic immunosuppression, and effects on survival and time to treatment failure in patients with melanoma treated with ipilimumab at Memorial Sloan Kettering Cancer Center. Journal of Clinical Oncology.

[94] Marthey L, Mateus C, Mussini C, Nachury M, Nancey S, Grange F, Zallot C, Peyrin-Biroulet L, Rahier JF, Bourdier de Beauregard M, Mortier L, Coutzac C, Soularue E, Lanoy E, Kapel N, Planchard D, Chaput N, Robert C, Carbonnel F (2016). Cancer immunotherapy with anti-CTLA-4 monoclonal antibodies induces an inflammatory bowel disease. Journal of Crohn's and Colitis.

[95] Lord JD, Hackman RC, Moklebust A, Thompson JA, Higano CS, Chielens D, Steinbach G, McDonald GB (2010). Refractory colitis following anti-CTLA4 antibody therapy: Analysis of mucosal FOXP3þ T cells. Digestive Diseases and Sciences.

[96] Robert C, Ribas A, Wolchok JD, Hodi FS, Hamid O, Kefford R, Weber JS, Joshua AM, Hwu WJ, Gangadhar TC, Patnaik A, Dronca R, Zarour H, Joseph RW, Boasberg P, Chmielowski B, Mateus C, Postow MA, Gergich K, Elassaiss-Schaap J, Li XN, Iannone R, Ebbinghaus SW, Kang SP, Daud A (2014). Anti-programmed-death-receptor-1 treatment with pembrolizumab in ipilimumab-refractory advanced melanoma: A randomised dose-comparison cohort of a phase 1 trial. The Lancet.

[97] Hughes MS, Zheng H, Zubiri L, Molina GE, Chen ST, Mooradian MJ, Allen IM, Reynolds KL, Dougan M (2019). Colitis after checkpoint blockade: A retrospective cohort study of melanoma patients requiring admission for symptom control. Cancer Medicine.

[98] Ribas A, Hodi FS, Callahan M, Konto C, Wolchok J (2018). Hepatotoxicity with combination of vemurafenib and ipilimumab. The New England Journal of Medicine.

[99] Reynolds K, Thomas M, Dougan M (2018). Diagnosis and management of hepatitis in patients on checkpoint blockade. ONCOLOGIST.

[100] Cramer P, Bresalier RS (2017). Gastrointestinal and hepatic complications of immune checkpoint inhibitors. Current Gastroenterology Reports.

[101] D’Angelo SP, Larkin J, Sosman JA, Lebbé C, Brady B, Neyns B, Schmidt H, Hassel JC, Hodi FS, Lorigan P, Savage KJ, Miller WH, Mohr P, Marquez-Rodas I, Charles J, Kaatz M, Sznol M, Weber JS, Shoushtari AN, Ruisi M, Jiang J, Wolchok JD (2016). Efficacy and safety of nivolumab alone or in combination with ipilimumab in patients with mucosal melanoma: A pooled analysis. Journal of Clinical Oncology.

[102] Garon EB, Rizvi NA, Hui R, Leighl N, Balmanoukian AS, Eder JP, Patnaik A, Aggarwal C, Gubens M, Horn L, Carcereny E, Ahn MJ, Felip E, Lee JS, Hellmann MD, Hamid O, Goldman JW, Soria JC, Dolled-Filhart M, Rutledge RZ, Zhang J, Lunceford JK, Rangwala R, Lubiniecki GM, Roach C, Emancipator K, Gandhi L (2015). Pembrolizumab for the treatment of non-small-cell lung cancer. The New England Journal of Medicine.

[103] Chuzi S, Tavora F, Cruz M, Costa R, Chae YK, Carneiro BA, Giles FJ (2017). Clinical features, diagnostic challenges, and management strategies in checkpoint inhibitor-related pneumonitis. Cancer Management and Research.

[104] Ma K, Lu Y, Jiang S, Tang J, Li X, Zhang Y (2018). The relative risk and incidence of immune checkpoint inhibitors related pneumonitis in patients with advanced cancer: A meta-analysis. Frontiers in Pharmacology.

[105] Cortellini A, Chiari R, Ricciuti B, Metro G, Perrone F, Tiseo M, Bersanelli M, Bordi P, Santini D, Giusti R, Grassadonia A, di Marino P, Tinari N, de Tursi M, Zoratto F, Veltri E, Malorgio F, Garufi C, Russano M, Anesi C, Zeppola T, Filetti M, Marchetti P, Berardi R, Rinaldi S, Tudini M, Silva RR, Pireddu A, Atzori F, Iacono D, Migliorino MR, Porzio G, Cannita K, Ficorella C, Buti S (2019). Correlations between the immune-related adverse events spectrum and efficacy of anti-PD1 immunotherapy in NSCLC patients. Clinical Lung Cancer.

[106] Michot JM, Bigenwald C, Champiat S, Collins M, Carbonnel F, Postel-Vinay S, Berdelou A, Varga A, Bahleda R, Hollebecque A, Massard C, Fuerea A, Ribrag V, Gazzah A, Armand JP, Amellal N, Angevin E, Noel N, Boutros C, Mateus C, Robert C, Soria JC, Marabelle A, Lambotte O (2016). Immune-related adverse events with immune checkpoint blockade: A comprehensive review. European Journal of Cancer.

[107] Abdel-Wahab N, Suarez-Almazor ME (2019). Frequency and distribution of various rheumatic disorders associated with checkpoint inhibitor therapy. Rheumatology (Oxford).

[108] Kostine M, Truchetet ME, Schaeverbeke T (2019). Clinical characteristics of rheumatic syndromes associated with checkpoint inhibitors therapy. Rheumatology (Oxford, England).

[109] Cappelli LC, Gutierrez AK, Bingham CO, Shah AA (2017). Rheumatic and musculoskeletal immune-related adverse events due to immune checkpoint inhibitors: A systematic review of the literature. Arthritis Care & Research (Hoboken).

[110] Warner, B. M., Baer, A. N., Lipson, E. J., Allen, C., Hinrichs, C., Rajan, A., Pelayo, E., Beach, M., Gulley, J. L., Madan, R. A., Feliciano, J., Grisius, M., Long, L., Powers, A., Kleiner, D. E., Cappelli, L., & Alevizos, I. (2019). Sicca syndrome associated with immune checkpoint inhibitor therapy. *ONCOLOGIST, 24*(9), 1259–1269. 10.1634/theoncologist.2018-0823. 10.1634/theoncologist.2018-0823 PMC6738284 30996010

[111] Cappelli, L. C., Gutierrez, A. K., Baer, A. N., Albayda, J., Manno, R. L., Haque, U., Lipson, E. J., Bleich, K. B., Shah, A. A., Naidoo, J., Brahmer, J. R., le, D., & Bingham III, C. O. (2017). Inflammatory arthritis and sicca syndrome induced by nivolumab and ipilimumab. *Annals of the Rheumatic Diseases, 76*(1), 43–50. 10.1136/annrheumdis-2016-209595. 10.1136/annrheumdis-2016-209595 PMC5333990 27307501

[112] Chan, K. K., & Bass, A. R. (2020). Autoimmune complications of immunotherapy: Pathophysiology and management. *The BMJ, 369*, m736. 10.1136/bmj.m736. 10.1136/bmj.m736 32253223

[113] Shingarev R, Glezerman IG (2019). Kidney complications of immune checkpoint inhibitors: A review. American Journal of Kidney Diseases.

[114] Abdel-Wahab N, Shah M, Suarez-Almazor ME (2016). Adverse events associated with immune checkpoint blockade in patients with cancer: A systematic review of case reports. PLoS One.

[115] Vandiver JW, Singer Z, Harshberger C (2016). Severe hyponatremia and immune nephritis following an initial infusion of nivolumab. Targeted Oncology.

[116] Shirali AC, Perazella MA, Gettinger S (2016). Association of acute interstitial nephritis with programmed cell death 1 inhibitor therapy in lung cancer patients. American Journal of Kidney Diseases.

[117] Hamid O, Robert C, Daud A, Hodi FS, Hwu WJ, Kefford R, Wolchok JD, Hersey P, Joseph RW, Weber JS, Dronca R, Gangadhar TC, Patnaik A, Zarour H, Joshua AM, Gergich K, Elassaiss-Schaap J, Algazi A, Mateus C, Boasberg P, Tumeh PC, Chmielowski B, Ebbinghaus SW, Li XN, Kang SP, Ribas A (2013). Safety and tumor responses with lambrolizumab (anti-PD-1) in melanoma. The New England Journal of Medicine.

[118] Cortazar FB, Marrone KA, Troxell ML, Ralto KM, Hoenig MP, Brahmer JR, le DT, Lipson EJ, Glezerman IG, Wolchok J, Cornell LD, Feldman P, Stokes MB, Zapata SA, Hodi FS, Ott PA, Yamashita M, Leaf DE (2016). Clinicopathological features of acute kidney injury associated with immune checkpoint inhibitors. Kidney International.

[119] Perazella MA, Shirali AC (2020). Immune checkpoint inhibitor nephrotoxicity: What do we know and what should we do?. Kidney International.

[120] Mahmood SS, Fradley MG, Cohen JV, Nohria A, Reynolds KL, Heinzerling LM, Sullivan RJ, Damrongwatanasuk R, Chen CL, Gupta D, Kirchberger MC, Awadalla M, Hassan MZO, Moslehi JJ, Shah SP, Ganatra S, Thavendiranathan P, Lawrence DP, Groarke JD, Neilan TG (2018). Myocarditis in patients treated with immune checkpoint inhibitors. Journal of the American College of Cardiology.

[121] Pradhan R, Nautiyal A, Singh S (2019). Diagnosis of immune checkpoint inhibitor-associated myocarditis: A systematic review. International Journal of Cardiology.

[122] Nishimura H, Okazaki T, Tanaka Y, Nakatani K, Hara M, Matsumori A, Sasayama S, Mizoguchi A, Hiai H, Minato N, Honjo T (2001). Autoimmune dilated cardiomyopathy in PD-1 receptor-deficient mice. Science.

[123] Varricchi G, Galdiero MR, Marone G, Criscuolo G, Triassi M, Bonaduce D, Marone G, Tocchetti CG (2017). Cardiotoxicity of immune checkpoint inhibitors. ESMO Open.

[124] Michel L, Rassaf T, Totzeck M (2019). Cardiotoxicity from immune checkpoint inhibitors. International Journal of Cardiology Heart & Vasculture.

[125] Lyon, A. R., Yousaf, N., Battisti, N. M. L., Moslehi, J., & Larkin, J. (2018). Immune checkpoint inhibitors and cardiovascular toxicity. *The Lancet Oncology, 19*(9), e447–e458. 10.1016/s1470-2045(18)30457-1. 10.1016/S1470-2045(18)30457-1 30191849

[126] Pirozzi, F., Poto, R., Aran, L., Cuomo, A., Galdiero, M. R., Spadaro, G., Abete, P., Bonaduce, D., Marone, G., Tocchetti, C. G., Varricchi, G., & Mercurio, V. (2021). Cardiovascular toxicity of immune checkpoint inhibitors: Clinical risk factors. *Current Oncology Reports, 23*(2), 13. 10.1007/s11912-020-01002-w. 10.1007/s11912-020-01002-w PMC7790474 33415405

[127] Salem, J. E., Manouchehri, A., Moey, M., Lebrun-Vignes, B., Bastarache, L., Pariente, A., Gobert, A., Spano, J. P., Balko, J. M., Bonaca, M. P., Roden, D. M., Johnson, D. B., & Moslehi, J. J. (2018). Cardiovascular toxicities associated with immune checkpoint inhibitors: An observational, retrospective, pharmacovigilance study. *The Lancet Oncology, 19*(12), 1579–1589. 10.1016/s1470-2045(18)30608-9. 10.1016/S1470-2045(18)30608-9 PMC6287923 30442497

[128] Chen, D. Y., Huang, W. K., Chien-Chia Wu, V., Chang, W. C., Chen, J. S., Chuang, C. K., & Chu, P. H. (2020). Cardiovascular toxicity of immune checkpoint inhibitors in cancer patients: A review when cardiology meets immuno-oncology. *Journal of the Formosan Medical Association, 119*(10), 1461–1475. 10.1016/j.jfma.2019.07.025. 10.1016/j.jfma.2019.07.025 31444018

[129] Hu JR, Florido R, Lipson EJ, Naidoo J, Ardehali R, Tocchetti CG, Lyon AR, Padera RF, Johnson DB, Moslehi J (2019). Cardiovascular toxicities associated with immune checkpoint inhibitors. Cardiovascular Research.

[130] Belum VR, Benhuri B, Postow MA, Hellmann MD, Lesokhin AM, Segal NH, Motzer RJ, Wu S, Busam KJ, Wolchok JD, Lacouture ME (2016). Characterisation and management of dermatologic adverse events to agents targeting the PD-1 receptor. European Journal of Cancer.

[131] Kamińska-Winciorek G, Cybulska-Stopa B, Lugowska I, Ziobro M, Rutkowski P (2019). Principles of prophylactic and therapeutic management of skin toxicity during treatment with checkpoint inhibitors. Advances in Dermatology and Allergology.

[132] Larkin J, Hodi FS, Wolchok JD (2015). Combined nivolumab and ipilimumab or monotherapy in untreated melanoma. The New England Journal of Medicine.

[133] Sibaud V (2018). Dermatologic reactions to immune checkpoint inhibitors : Skin toxicities and immunotherapy. American Journal of Clinical Dermatology.

[134] Boutros C, Tarhini A, Routier E, Lambotte O, Ladurie FL, Carbonnel F, Izzeddine H, Marabelle A, Champiat S, Berdelou A, Lanoy E, Texier M, Libenciuc C, Eggermont AMM, Soria JC, Mateus C, Robert C (2016). Safety profiles of anti-CTLA-4 and anti-PD-1 antibodies alone and in combination. Nature Reviews. Clinical Oncology.

[135] Hua C, Boussemart L, Mateus C, Routier E, Boutros C, Cazenave H, Viollet R, Thomas M, Roy S, Benannoune N, Tomasic G, Soria JC, Champiat S, Texier M, Lanoy E, Robert C (2016). Association of vitiligo with tumor response in patients with metastatic melanoma treated with pembrolizumab. JAMA Dermatology.

[136] Freeman-Keller M, Kim Y, Cronin H, Richards A, Gibney G, Weber JS (2016). Nivolumab in resected and unresectable metastatic melanoma: Characteristics of immune-related adverse events and association with outcomes. Clinical Cancer Research.

[137] Sibaud V, Meyer N, Lamant L, Vigarios E, Mazieres J, Delord JP (2016). Dermatologic complications of anti-PD-1/PD-L1 immune checkpoint antibodies. Current Opinion in Oncology.

[138] Liao B, Shroff S, Kamiya-Matsuoka C, Tummala S (2014). Atypical neurological complications of ipilimumab therapy in patients with metastatic melanoma. Neuro-Oncology.

[139] Cuzzubbo, S., Javeri, F., Tissier, M., Roumi, A., Barlog, C., Doridam, J., Lebbe, C., Belin, C., Ursu, R., & Carpentier, A. F. (2017). Neurological adverse events associated with immune checkpoint inhibitors: Review of the literature. *European Journal of Cancer, 73*, 1–8. 10.1016/j.ejca.2016.12.001. 10.1016/j.ejca.2016.12.001 28064139

[140] Wick, W., Hertenstein, A., & Platten, M. (2016). Neurological sequelae of cancer immunotherapies and targeted therapies. *The Lancet Oncology, 17*(12), e529–e541. 10.1016/s1470-2045(16)305771-x. 10.1016/S1470-2045(16)30571-X 27924751

[141] Johnson, D. B., Wallender, E. K., Cohen, D. N., Likhari, S. S., Zwerner, J. P., Powers, J. G., Shinn, L., Kelley, M. C., Joseph, R. W., & Sosman, J. A. (2013). Severe cutaneous and neurologic toxicity in melanoma patients during vemurafenib administration following anti-PD-1 therapy. *Cancer Immunology Research, 1*(6), 373–377. 10.1158/2326-6066.cir-13-0092. 10.1158/2326-6066.CIR-13-0092 PMC3905602 24490176

[142] Dalvin LA, Shields CL, Orloff M, Sato T, Shields JA (2018). Checkpoint inhibitor immune therapy: Systemic indications and ophthalmic side effects. Retina-The Journal Of Retinal And Vitreous Diseases.

[143] Haanen JBAG, Carbonnel F, Robert C, Kerr KM, Peters S, Larkin J, Jordan K, ESMO Guidelines Committee (2017). Management of toxicities from immunotherapy: ESMO Clinical Practice Guidelines for diagnosis, treatment and follow-up. Annals of Oncology.

[144] Postow MA, Sidlow R, Hellmann MD (2018). Immune-related adverse events associated with immune checkpoint blockade. The New England Journal of Medicine.

[145] Postow MA, Chesney J, Pavlick AC, Robert C, Grossmann K, McDermott D, Linette GP, Meyer N, Giguere JK, Agarwala SS, Shaheen M, Ernstoff MS, Minor D, Salama AK, Taylor M, Ott PA, Rollin LM, Horak C, Gagnier P, Wolchok JD, Hodi FS (2015). Nivolumab and ipilimumab versus ipilimumab in untreated melanoma. The New England Journal of Medicine.

